# Radiation Type- and Dose-Specific Transcriptional Responses across Healthy and Diseased Mammalian Tissues

**DOI:** 10.3390/antiox11112286

**Published:** 2022-11-18

**Authors:** Eftychia Sagkrioti, Gökay Mehmet Biz, Işıl Takan, Seyedehsadaf Asfa, Zacharenia Nikitaki, Vassiliki Zanni, Rumeysa Hanife Kars, Christine E. Hellweg, Edouard I. Azzam, Stella Logotheti, Athanasia Pavlopoulou, Alexandros G. Georgakilas

**Affiliations:** 1 DNA Damage Laboratory, Physics Department, School of Applied Mathematical and Physical Sciences, National Technical University of Athens (NTUA), Zografou, 15780 Athens, Greece; 2 Biology Department, National and Kapodistrian University of Athens (NKUA), 15784 Athens, Greece; 3 Department of Technical Programs, Izmir Vocational School, Dokuz Eylül University, Buca, Izmir 35380, Turkey; 4 Izmir Biomedicine and Genome Center (IBG), Balcova, Izmir 35340, Turkey; 5 Izmir International Biomedicine and Genome Institute, Dokuz Eylül University, Balcova, Izmir 35340, Turkey; 6 Department of Biomedical Engineering, Istanbul Medipol University, Istanbul 34810, Turkey; 7 German Aerospace Center (DLR), Institute of Aerospace Medicine, Radiation Biology, Linder Höhe, D-51147 Köln, Germany; 8 Canadian Nuclear Laboratories, Chalk River, ON K0J 1J0, Canada

**Keywords:** radiation response, bioinformatics, oxidative stress, transcriptomics, radiobiology database, gene signature

## Abstract

Ionizing radiation (IR) is a genuine genotoxic agent and a major modality in cancer treatment. IR disrupts DNA sequences and exerts mutagenic and/or cytotoxic properties that not only alter critical cellular functions but also impact tissues proximal and distal to the irradiated site. Unveiling the molecular events governing the diverse effects of IR at the cellular and organismal levels is relevant for both radiotherapy and radiation protection. Herein, we address changes in the expression of mammalian genes induced after the exposure of a wide range of tissues to various radiation types with distinct biophysical characteristics. First, we constructed a publicly available database, termed RadBioBase, which will be updated at regular intervals. RadBioBase includes comprehensive transcriptomes of mammalian cells across healthy and diseased tissues that respond to a range of radiation types and doses. Pertinent information was derived from a hybrid analysis based on stringent literature mining and transcriptomic studies. An integrative bioinformatics methodology, including functional enrichment analysis and machine learning techniques, was employed to unveil the characteristic biological pathways related to specific radiation types and their association with various diseases. We found that the effects of high linear energy transfer (LET) radiation on cell transcriptomes significantly differ from those caused by low LET and are consistent with immunomodulation, inflammation, oxidative stress responses and cell death. The transcriptome changes also depend on the dose since low doses up to 0.5 Gy are related with cytokine cascades, while higher doses with ROS metabolism. We additionally identified distinct gene signatures for different types of radiation. Overall, our data suggest that different radiation types and doses can trigger distinct trajectories of cell-intrinsic and cell-extrinsic pathways that hold promise to be manipulated toward improving radiotherapy efficiency and reducing systemic radiotoxicities.

## 1. Introduction

Radiation therapy has witnessed unprecedented advances during the last decades, asserting its place as a major part of everyday clinical practice [1]. It contributes to ~40% of curative cancer treatments [2], alone or in combination with chemotherapy [3], and tends to be less morbid than surgery [4]. In addition to its direct cytotoxic effects on the targeted tumors, irradiation often triggers indirect localized and systemic responses. These responses are not only occasionally linked with early or late adverse side effects proximal or distal to the treatment site but can also be beneficial for patient outcomes. Intriguingly, recent studies show that radiotherapy induces *bona fide* immunogenic cell death and engages tumor-targeting immune responses in support of enhancing treatment efficacy. Local irradiation reshapes the tumor microenvironment (TME) by promoting prooxidant and proinflammatory reactions, which may trigger adaptive immune responses [1]. Stressed and dying irradiated cells release numerous bioactive molecules, for example, major histocompatibility complex, cell-adhesion molecules, and proinflammatory cytokines and their receptors, as well as molecules with damage-associated molecular patterns (DAMPs), small metabolites, nucleic acids and lipids. These tumor-associated antigens interact with the immune system to induce immunogenic cell death [5,6] since they are taken up by the dendritic cells and stimulate downstream effector T cells, which subsequently recognize and lyse tumor cells both locally and at distant sites [7]. In several clinical cases, tumors distal to the targeted site regressed in response to irradiation-induced immunogenicity, a phenomenon termed as an abscopal effect [7]. In this respect, the irradiated cells act as in situ vaccines against tumors, sensitizing the immune system to detect cancer cells even long after the completion of radiation treatment. Hence, systemic effects of radiotherapy may act as a ”blessing in disguise” due to their potential to ally with the immune system and increase responses that control the growth of micrometastases and malignant tissues at distant sites. However, the effects may also be a “curse” resulting in the suppression of antitumor immunity by mechanisms involving regulatory T cells [8].

The newly-discovered immunomodulatory properties of radiation have been linked with its ability to primarily activate the DNA damage response and repair (DDR/R) machinery. DDR/R is a highly conserved and complex network of signal transduction pathways that preserves the genetic information by repairing a variety of DNA lesions, such as nucleotide alterations, bulky adducts, single-strand breaks (SSBs) and double-strand breaks (DSBs). These pathways can be lesion-specific, for example, non-homologous end joining (NHEJ) and homologous recombination (HR) repair for DSBs; single-strand break repair (SSBR) for nicked DNA strands; mismatch repair (MMR) for errors that occurred during replication; base excision repair (BER) for oxidative base modifications; and nucleotide excision repair (NER) for helix-distorting lesions [9]. The stimulation of different components of DDR/R, either endogenously or from external sources, such as exposure to ionizing radiation (IR), alerts host immunity at the systemic level and vice versa [10], thereby accounting for the intriguing immunogenic properties of irradiated cells. These novel concepts have rejuvenated clinical interest to exploit this dynamic and bidirectional crosstalk between DDR/R and immune response (ImmR) signaling and manipulate it towards personalized radiotherapeutic solutions.

There are several types of therapeutic modalities, classified according to radiation quality associated mainly with the linear energy transfer (LET), a parameter accounting for the amount of energy deposited per unit length of the irradiating particle’s path. Low-LET radiation entails the more frequently used γ- and X-rays, while high-LET refers to protons, carbon ions and α-particles that capitalize on the physical and radiobiological properties of charged particles for an improved dose distribution and increased cell killing efficacy. Carbon ions kill cells twice or three times more effectively than protons and conventional radiation modalities [11]. In general, high-LET types induce more DSBs per dose unit, and more complex and dense lesions than low-LET types because they deposit large amounts of energy within a small distance [12]. The type of initial DNA damage largely determines the repair pathway that is subsequently activated. For example, heavy ions preferentially shift towards DSB repair pathways, such as HR and NHEJ, when compared with sparsely ionizing irradiation [1]. Given that a different type of DNA damage can trigger different DDR components, which in turn are associated with the release of immunostimulatory neoantigens as “danger signals” (i.e., DAMPs), it is reasonable to envisage that each of these irradiation types governs distinct trajectories of DNA damage type—DDR pathway—immunogenic responses, which, however, to date, have not been identified [1]. In this regard, understanding the major differences of low- and high-LET treatment options is a current challenge of radiotherapy, not only for minimizing side effects, but also for making the most of each modality toward stimulating tumor-targeting adaptive immunity post-irradiation. 

The effects of the various radiation types are mediated, at least partly, through changes in the transcriptomes of the irradiated cells. In general, different types of radiation trigger distinct gene transcription programs associated with divergent cellular responses both in cancer and normal cells. Although radiation type-specific transcriptional changes have been examined sporadically [13,14,15,16,17,18], to our knowledge, there is no systematic effort to characterize the effects of several high-LET or low-LET radiation types and doses of radiation in normal or diseased tissues, which would set a basis to untangle their side effects from their beneficial cytotoxic and immunogenic properties. Simultaneous screening of the transcriptomes across irradiated cancer and normal tissues would require large-scale experiments for each radiation type and/or dose. Furthermore, due to the genetic heterogeneity of cells in irradiated tissues, which is a major parameter of the efficacy of radiotherapy, extensive testing on a large variety of tissue contexts is required, transforming this effort to a “Herculean task”.

As a “*deus ex machina*”, computational approaches have entered the stage of radiobiology to accelerate and complement these efforts. In the present work, we constructed a publicly available, user-friendly database, termed RadBioBase version 1 (http://radbiodb.physics.ntua.gr/), which includes a collection of up-to-date existing data on mammalian genes differentially expressed after exposure to different types (X-rays, γ-rays, protons, carbon ions and α-particles) and doses of radiation in a variety of cell types. This database is a comprehensive tool for correlations of radiation type and/or dose with corresponding transcriptional responses across a variety of tissues. Following an integrated bioinformatics approach that included gene-centric, pathway-oriented and machine learning analyses, we consolidated the IR-induced differential gene expression to biological pathways and human diseases. In addition, we identified gene signatures for different radiation types. Our analyses provide insights into the links between the IR-induced damage and the signal propagation of stress to distant sites, and hold promise for a deeper understanding of the association between DDR and the immune system to a wider context, in a coordinated multiscale manner, which could be translated to more efficient and safer radiotherapy schemes.

## 2. Materials and Methods

### 2.1. Data Hybrid Collection and Transcriptomic Analyses

A broad collection of genes was initially obtained by rigorous text mining of the bibliographic database MEDLINE/PubMed 2.0 (https://pubmed.ncbi.nlm.nih.gov/, accessed on 15 March 2021), with the use of keywords related to X-ray, γ-ray, proton, carbon ion and α-particle irradiation, i.e., ((gamma radiation) OR (gamma rays) OR (γ rays)) AND gene expression; ((proton(Title/Abstract)) AND (radiation(Title/Abstract))) AND (gene expression(Title/Abstract)); ((carbon(Title/Abstract)) AND (radiation(Title/Abstract))) AND (gene expression(Title/Abstract)) from 1 January 2006 to 30 August 2021. The articles were independently retrieved from the literature by three of the authors (E.S, R.H.K. and A.P.). Relevant data were extracted from the articles and recorded into an Excel worksheet.

For the articles to be considered eligible for inclusion in our study, they had to report the following information: (i) tissue/cell line, (ii) cell type (cancer or normal), (iii) model organism, (iv) type of irradiation, (v) irradiation exposure time, (vi) dose amount, (vii) availability of data regarding genes differentially expressed between irradiated and non-irradiated (control) cells/tissues, or sufficient data to calculate differential gene expression. To minimize investigator biases, compliance of the screened articles with the study eligibility criteria was assessed, independently, by three researchers, E.S, R.H.K. and A.P. and validated by the supervising researcher (A.G.G.). In this way, a total of 39 studies were selected. Gene symbols were assigned to the extracted human, mouse and rat genes according to the HUGO Gene Nomenclature Committee (HGNC) (https://www.genenames.org/, accessed on 20 November 2021).

In cases where differential gene expression data were not provided in the corresponding articles, we searched for the original gene expression data files deposited in NCBI GEO (Gene Expression Omnibus) DataSets [19] according to the selection criteria: (i) gene expression data derived from irradiated and non-irradiated (control) tissue/cell samples, and (ii) inclusion of >5000 genes in the dataset. The following microarray transcriptome datasets were obtained where their respective GEO series and PubMed references are shown in brackets: X-rays (GSE107685 [20], GSE113611 [21], GSE107443 [22], GSE90909 [23], GSE85323 [24], GSE59861 [25], GSE6262 [26]); α particles (GSE12435 [27], GSE21059 [28], GSE18760 [29]); carbon ions (GSE6630 [30]); protons (GSE20629 [31]). The GEO2R interactive web server [19] was employed to detect genes differentially expressed at different conditions. 

The differentially expressed genes (DEGs) with an absolute log2 fold-change (FC) greater than 1.5 (|log2FC ≥ 1.5|), or FC > 1.5 and FC < 0.67, and FDR-adjusted *p*-value (q-value) less than 0.05 or *p*-value < 0.001 (for transcriptomic data) and *p*-value < 0.05 (for the text mining data) were retained.

### 2.2. Functional Enrichment Analysis

Venn diagrams were constructed using the online tool Draw Venn Diagram (https://bioinformatics.psb.ugent.be/webtools/Venn/, accessed on 20 January 2022) to identify common up and downregulated genes across radiation types, as well as of low- versus high-LET radiation (only for entries where the corresponding LET was provided in the original paper) and deregulated genes of lower versus higher doses in the range of clinical interest (0.3–0.5 Gy vs. 0.6–2.0 Gy). Furthermore, overrepresented biological pathways, along with the corresponding disease pathways, were identified in different sets of genes, related to every type of irradiation, as well as for low and high LET, and low and high clinical doses. Functional enrichment analysis was conducted with WebGestalt (WEB-based GEne SeT AnaLysis Toolkit) 2019, an online tool used for the identification of statistically significant enriched terms in the given gene sets compared to selected reference sets [32]. The WebGestalt parameters chosen were “Organism of Interest”: Homo sapiens, “Method of Interest”: Over-Representation Analysis (ORA), “Functional database”: geneontology/Biological Process noRedundant and pathway/Wikipathway for biological paths, or disease/Disgenet for diseases, “Select gene ID type”: gene symbol, “Select Reference set”: genome; the default advanced parameters were chosen, and only pathways with false discovery rate (FDR)-corrected *p*-value less than 0.05 were considered in the analysis. Affinity propagation was used for clustering the terms (i.e., biological process and disease) by selecting a subset of representative terms.

### 2.3. Database Construction

API-based Directus (https://docs.directus.io/, accessed on 10 April 2022), an open-source data platform, was used for content management, and MySQL (https://dev.mysql.com/, accessed on 10 April 2022), an open-access database management system, was used to store the data on the backend side. Data stored in excel format were imported to the MySQL database using Node.js.

On the front end, the popular VueJS framework, which provides officially maintained support packages for building web UIs, was used to create easily accessible content interfaces. Axios library (https://axios-http.com/, accessed on 12 April 2022), a promise-based HTTP client for the browser and Node.js, was used to obtain the data provided by Directus content management API services. Tailwind CSS framework (https://tailwindcss.com/, accessed on 12 April 2022) was utilized for the styles of the website interface.

### 2.4. Machine Learning Approach

Random Forest is a bagging ensemble algorithm, which uses multiple different algorithms to generate a consensus output. It accepts as input a random sample generated from a given dataset with replacement, and then this sample is fed into the tree classifiers. At the end, the class of the sample is determined by voting with the principle of majority rule. During data classification, it can also provide the importance score of each variable (e.g., gene) and evaluate its role in the classification. There are many popular methods for gene selection, including deep gene selection [33], WERFE [34], Based Bayes error Filter [35], etc. The basic principle of all of these methods is to firstly rank the genes on the basis of certain evaluation criteria, and then select an optimal subset of genes. However, these methods cannot capture the relationship between the selected genes and the precision of the classification. Su and colleagues developed an algorithm based on recursive feature elimination (RFE), by taking into account the impact of both the gene numbers and prediction performance [36].

RFE is a greedy algorithm that creates gene sets recursively and then determines an optimal subset from those sets. The goal of RFE is to obtain the smallest possible sets of variables in an iterative way. RFE discards those genes of least importance in an iterative way and performs classification based on the new subsets of genes. All the gene subsets are evaluated based on their classification performance. 

In our study, in order to prioritize the genes in the groups (a) irradiated *versus* non-irradiated, and (b) cancer *versus* normal, we first applied the RFE algorithm in Random Forest. All the methods were implemented by using the Python 3.9.7 *scikit-learn* module (https://pypi.org/project/scikit-learn/, accessed on 16 February 2022). To this end, we randomly divided our datasets into 75% training data and 25% testing data for all the models used for classification; the random state was set to 42. We first fit the model, then removed the less relevant genes (listed in the RadBioBase) and calculated the classification performance metric. After that, we removed the least important genes, fitted the model again and calculated the performance. This process was repeated until there were no genes left. The final set of genes was the set that maximized the performance. However, the gene subset selected in this study was the one with the highest accuracy since accuracy is the most common evaluation metric adopted for assessing the robustness and efficiency of algorithms. The final gene subsets of high *versus* low LET demonstrated classification accuracies of 95.54%, respectively.

Finally, to enhance the robustness of our results, robust rank aggregation (RRA) [37] was applied to the output of the previous steps so as to obtain the top-ranking genes. The RRA method uses a noise-robust probabilistic model to aggregate ranked lists, such as lists of genes, and to calculate the statistical significance (*p*-values) for all ranked elements. RRA was performed in the R programming environment (version 4.1.3) (https://www.r-project.org/, accessed on 10 March 2022).

### 2.5. Functional Network

The STRING database (version 11.5) (https://string-db.org/, accessed on 15 May 2022) [38] was used to investigate and visualize both known and predicted associations among the protein products of the genes under study.

## 3. Results and Discussion

### 3.1. Development of RadBioBase

For database construction, we performed text mining in PubMed, using appropriate keywords across studies that experimentally address the overall effect of five high- and low-LET radiation types of interest in a broad range of mammalian cell types, including human, mouse and rat study model systems. The database includes 7436 entries, with a total of 3730 unique genes derived from 14 tissues/cell lines [20,21,22,23,24,25,26,27,28,29,30,31,39,40,41,42,43,44,45,46,47,48,49,50,51,52,53,54,55,56,57,58,59,60,61,62,63,64,65]. For each entry, the following information was provided:

- Differential expression of genes: The expression status of the corresponding genes (i.e., up or downregulated in irradiated compared to non-irradiated tissue/cell control groups). In this version of the database, the canonical, full-length transcripts for each gene were used.
- Biological characteristics: Cell type (cancer or normal), organism and tissue/cell line.
- Type of irradiation: X-rays, γ-rays, protons, carbon ions or α-particles.
- Post-irradiation time when provided in the original study.
- Physical characteristics: LET (keV/μm), beam energy (MeV or kV for X-rays), dose (Gy) and average dose rate (Gy/min or Gy/h). In the cases where the LET of particles was not included in the original paper, it was calculated with the Stopping and Range of Ions in Matter (SRIM/TRIM) software, using as entrance parameters the type of ion, the target density and the energy of the irradiation beam when provided. For tissue targets not included in the compound dictionary of SRIM, the elemental compositions and mass densities were obtained from the bibliography [66,67,68]. Notably, the SRIM-calculated LET values were calculated only when provided in the relative studies, for the entrance point (highest energy values) of the beam instead of the Bragg peak, and thus were much smaller than the expected LET values for the Bragg peak region. According to the different energies in the various studies, LET values for protons were calculated as such: energies 100 MeV----> 0.76 keV/μm, 250 MeV----> 0.34 keV/μm, 190.6 MeV----> 0.5 keV/μm, 230 MeV----> 0.38 keV/μm, 4.5 MeV----> 9.54 keV/μm (Table S1). Moreover, those α-particle energies not provided in the original paper were calculated empirically with the help of LET-energy curves [69].
- Comparison with low-LET irradiation: X-rays, γ-rays or electrons, depending on the information given in the original paper.
- DNA damage (in clusters per Gy per Gbp): DSBs and total clusters of DNA damage were calculated using the Monte Carlo Damage Simulation (MCDS) software [70,71] for each radiation type (Table S1). For each MCDS input file, the parameters were set as CELL: DNA = 1 ndia = 5 cdia = 10, SIMCON: nocs = 10,000 seed = 987,654,321, and the oxygen concentration was set to 20%, while X-ray and γ-ray radiation was simulated by a 10 keV electron beam. The inclusion of the “complex damages” is based on the well-documented importance of clustered DNA damages in defying biological responses and can provide the first hints for possible connections of the quality and quantity of DNA damage with specific gene expression [72]. PubMed ID of the corresponding article.
- Type of validation: depending on the method used in the original studies for data validation, we defined values as (a) microarrays, (b) RNA-Seq, (c) qPCR, (d) microarrays and qPCR, and (e) RNA-Seq and qPCR.

**Figure 1:**
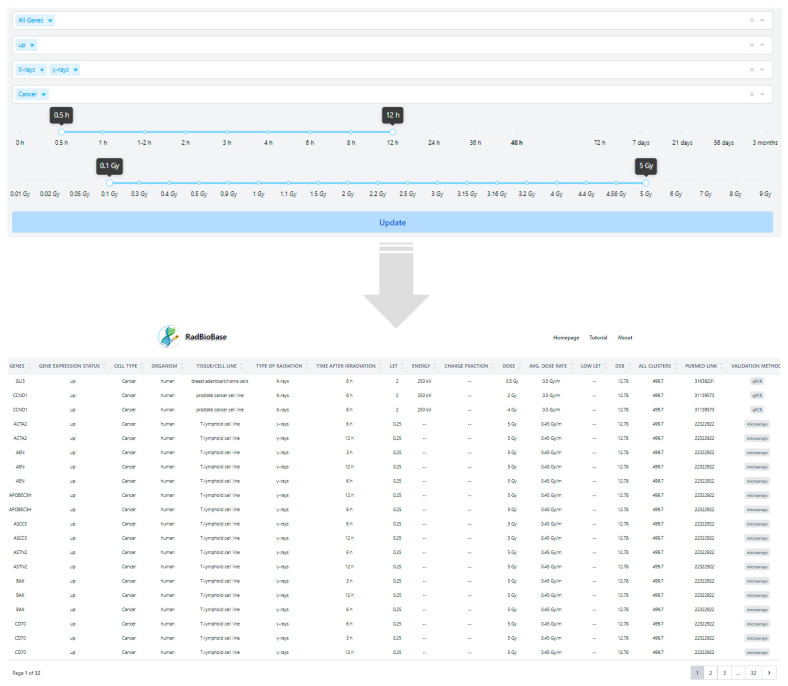
Example output page of RadBioBase. The database was searched using the “X-rays & γ-rays”, “Cancer” and “up” options, by selecting the time and dose ranges 0.5–-12 h and 0.1–5 Gy, respectively.

The above data are available through RadBioBase (http://radbiodb.physics.ntua.gr/). RadBioBase has a user-friendly interface and can be searched by using several options, such as (a) differentially expressed genes (b) gene expression status (up or downregulated), (c) type of radiation, (d) cell type (normal or cancer), (e) radiation dose, (f) radiation exposure time, (g) as well as a combination of the above options (Figure 1). The search results are displayed in a new window, in a tabular format, and can be downloaded to a CSV file. RadBioBase v1 is maintained by the National Technical University of Athens, Greece, and will be updated at regular intervals.

### 3.2. Commonalities among Radiation Types across a Number of Mammalian Tissues

**Figure 2:**
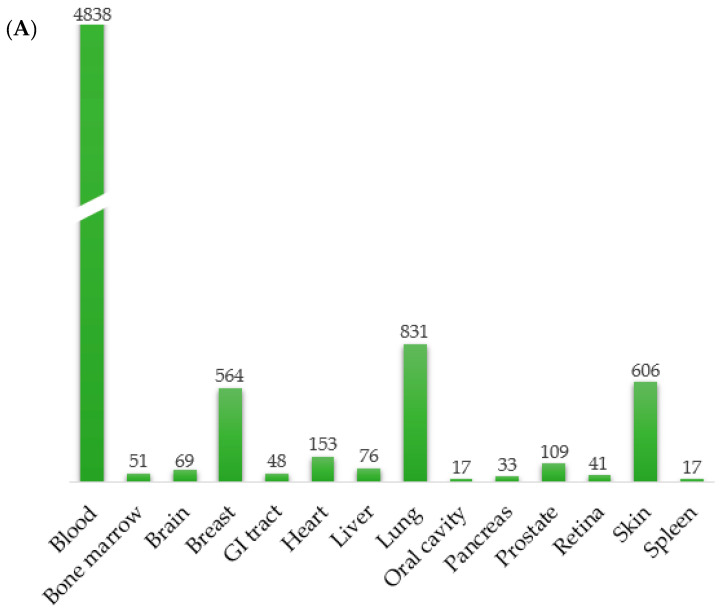

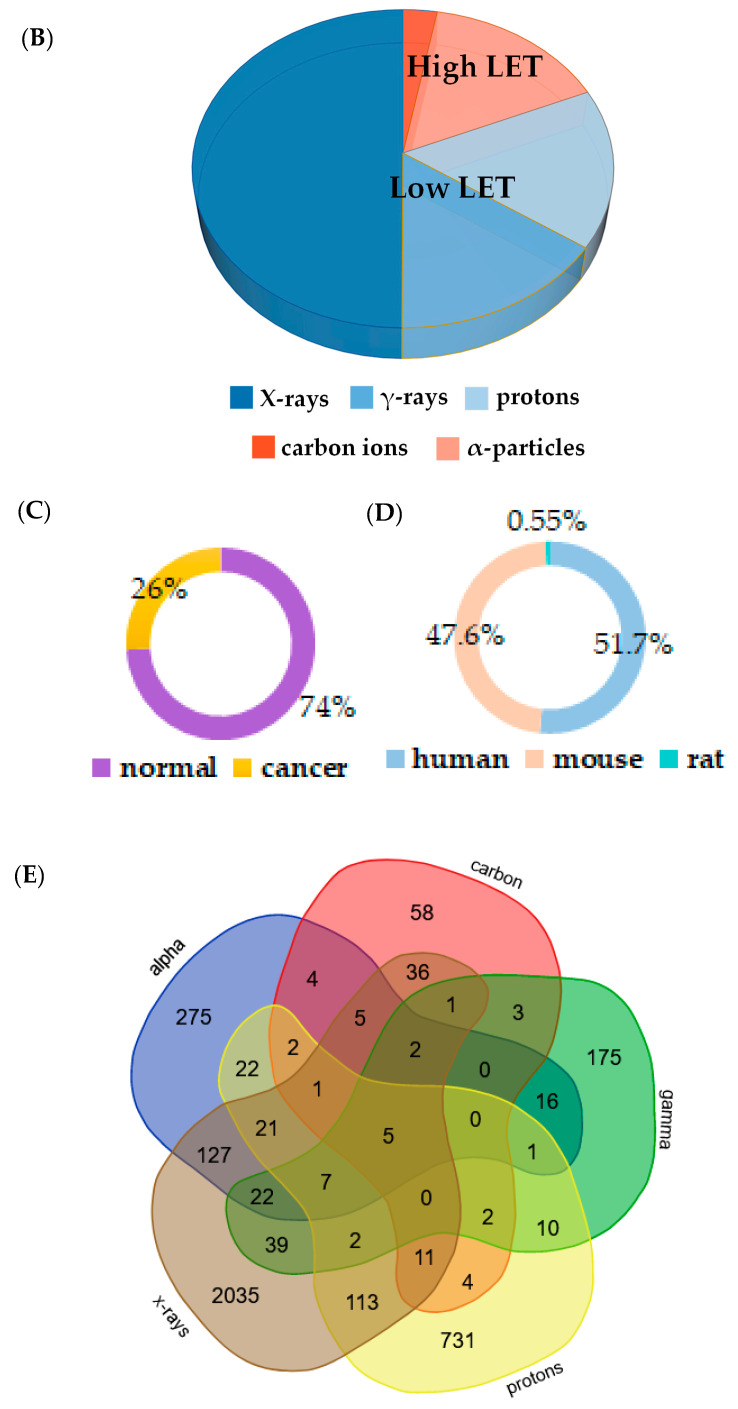
A description of the contents of the database and commonalities among the several radiation types. Database statistics. (**A**) Number of entries corresponding to tissues and cell lines are shown on top of the bars; the height of the bars is proportional to the number of entries. Percentage of entries related to (**B**) radiation types, (**C**) cancer and normal, (**D**) human and rodent tissues/cells across different types of radiation. (**E**) Venn diagram illustrating the overlapping of differentially expressed genes (both up and downregulated) between all radiation groups. The five common genes in all radiation groups are *GDF15*, *GADD45A*, *SESN1*, *CDKN1A* and *TP53INP1*.3.3. Each radiation type is linked to distinct biological functions.

Using RadBioBase, we performed a comparison among all different types of irradiation (X-rays, γ-rays, protons, carbon ions and α-particles), to unveil basic commonalities across all therapeutic modalities and mammalian tissue types. One important consideration regarding this database is that, since its generation is based on publicly available data, it is inevitably more representative for the types of tissues and irradiation most frequently used across the corresponding studies. To collectively describe the content of this database, we estimated the number of entries for tissue type, radiation type, organism type and normal versus cancer cell type (Figure 2A–D). Overall, the database includes 14 types of cells/tissues (Figure 2A). The highest number of entries are assigned to blood, breast and lung tissue, possibly reflecting the types of cancers where irradiation represents a frequent standard of care treatment. Similarly, 50% of the entries correspond to X-rays, which have been in research and clinical use for longer periods than the more recent radiation types (Figure 2B). Moreover, 74% of the entries represent normal and 26% cancer cells (Figure 2C). The percentages of entries in human versus rodent cells are similar, leading to a ratio of 1.06 (Figure 2D). This information facilitates the design of downstream analyses, interpretations of the results and inferences about disease pathways, especially in cases where the data are combined to generate universal signatures.

As shown in Figure 2E, among all up and downregulated genes (included in the current version of RadBioBase), we identified five genes that are commonly activated in all radiation groups (*GDF15*, *GADD45A*, *SESN1*, *CDKN1A* and *TP53INP1*). These genes are downstream effectors/targets of p53, a major tumor suppressor gene that encodes a transcription factor with a central role in preserving cell homeostasis and is one of the most important targets for translational cancer research. The physiologically low levels of mature p53 increase upon cellular stresses and, together with post-translational modifications, lead to the formation of oligomers that bind to specific p53 responsive elements on target gene promoters. Upon limited DNA damage, p53 induces cell cycle arrest and DNA repair genes, whereas upon extended and severe damage, it induces genes mediating senescence or cell death so as to isolate damaged cells from the intact cellular population [73]. The p53 pathways control five different kinds of cell death: (i) apoptosis, (ii) ferroptosis, (iii) TNF ligand- or (iv) FAS ligand-mediated necroptosis and (v) cellular senescence followed by the secretion of cytokines that attract immune system cells [74]. Our results are consistent with studies suggesting that the p53 pathway is a universally-induced sensitizer of cells to any type of irradiation [74]. They also suggest that p53-targeting molecules hold potential to be combined with any type of radiotherapeutic modality to increase treatment efficacy across a number of tissues. 

As shown in Figure 2E, the number of non-overlapping genes for each radiation type tends to be higher than the genes that are in common in two or more radiation types. The fact that transcriptional responses tend to be radiation type-specific strongly indicates that along with the p53 cascades, each radiation modality can activate distinct biological pathways to exert its effects on cells. In an analogous manner, radiation-specific transcripts might be associated with different disease pathways, which can be predictors of specific side effects. To shed light on these aspects, we performed a detailed analysis of the overrepresented biological and disease pathways related to each type of radiation separately, along with the corresponding genes.

**Figure 3:**
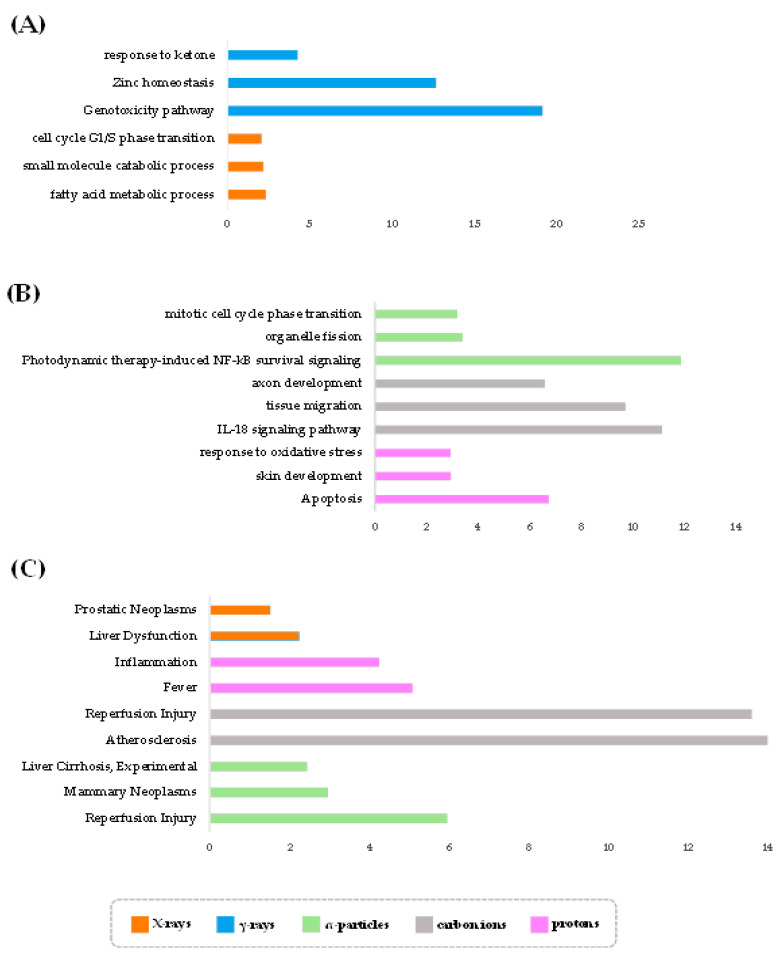
Gene set enrichment analysis. Overrepresented biological pathways (affinity propagation) for the DEGs in different types of radiation. (**A**) Low-LET radiation (X- and γ-rays) and (**B**) higher LET radiation (protons, carbon ions, α-particles). (**C**) Overrepresented disease pathways (affinity propagation) for all DEGs genes in different radiation types. The *x*-axis corresponds to the enrichment ratio, i.e., the ratio of the number of observed genes to the number of expected genes from each category in the input gene list.

We found that each radiation type, in general, exhibits a unique set of biological processes, beyond the expected pathways of response to stress and cell death. In particular, X-rays are related to metabolic processes, including the “fatty acid metabolic process”, “small molecule catabolic process” and “sulfur compound metabolic process” (Figure 3A and Table S2). This is consistent with several studies showing that IR can cause metabolic changes, oxidative stress and cell death [75,76] and that sulfur-related enzymes play a major role in the radiation-induced oxidative stress response and detoxification [77]. Upon irradiation, where the levels of oxygen-free radicals are increased, sulfur-related metabolism acts as an antioxidative stress defense pathway. These processes are particularly prominent in the liver since its function is critical in the protection against induced stress, rendering the liver extremely sensitive to radiation. X-ray irradiation was also found to be associated with fatty acid (FA) metabolism. Interestingly, recent studies suggest that FA metabolism represents the link between X-ray irradiation and ferroptosis, a novel type of programmed cell death that depends on iron and is characterized by the accumulation of lipid peroxides [78]. This FA-related type of cell death is genetically and biochemically distinct from other forms of regulated cell death. In agreement, ferroptosis-inducing agents can sensitize cancer cells to X-ray irradiation [79], while pro-ferroptotic FA metabolism renders cancer cells immunogenic [80]. In light of these data, it would be interesting to investigate whether X-rays initiate an FA metabolism–ferroptosis axis, which subsequently modulates the immunogenic properties of irradiated cells towards enhancing therapeutic responses to immunotherapy. 

Additionally, we found associations of X-ray-induced transcriptomes with zinc and copper homeostasis (Figure 3A and Table S2). On the one hand, zinc homeostasis is indirectly related to post-irradiation effects through increases in oxidative stress [81,82,83]. Zinc exhibits protective effects against irradiation by activating antioxidant enzymes, which in turn reduce reactive oxygen species (ROS) levels and oxidative stress [81,83,84]. In addition, zinc acts as an intracellular signaling molecule, activating apoptotic pathways, immunodeficiency and inflammation suppression [81,83,85]. On the other hand, copper ions contribute to radiation- and stress-resistance [86], tumor growth, inflammation and angiogenesis [87,88,89,90].

Among higher LET radiation types, protons are strongly related to apoptosis and oxidative stress (Figure 3B and Table S2), while carbon ion and alpha particles with enhanced proinflammatory signaling. However, while carbon ions exhibit overrepresented interleukin-18 (IL-18) signaling pathways (Figure 3B and Table S2), α-particles appear to be linked with photodynamic therapy (PDT)-induced NF-κB survival signaling (Figure 3B and Table S2). IL-18 is a proinflammatory cytokine of the interleukin-1 family, expressed in several cell types, including, but not limited to, macrophages, dendritic cells and epithelial cells. It is also involved in the regulation of immunomodulatory cytokine networks that mediate host defense, inflammation and tissue regeneration [91]. Regarding the transcription factor NF-κB, it integrates several stress signals and can regulate DNA transcription, cell survival, as well as immune system and inflammatory responses in a pleiotropic manner. NF-κB pathways are triggered by PDT and regulate the interplay between the immune system and an anti-cell death response through the release of cytokines and chemokines and the control of apoptosis or necrosis [92]. Intriguingly, IL-18 can also activate NF-κB; therefore, it is possible that the effects of carbon ions and alpha particles revolve around a complex inflammatory and immunomodulatory network, where NF-κB occupies a central hub position suggested also by Hellweg (2015) [93]. Taking into account that higher LET radiation can cause a higher level of DSBs and DNA damage clusters [94], it would be interesting to further investigate if these pathways may stand at the crossroads of high LET-specific DNA damage and the immune response [95,96]. 

We also observed that carbon ions activate transcripts involved in axon guidance and cell migration [96]. This finding is consistent with studies suggesting that cell migration and apoptosis in normal and tumorigenic tissues is regulated by many axon guidance molecules [97]. Notably, tumor-intrinsic activation of genes indispensable for neuronal development and neurological function is a nearly universal phenomenon in cancer, which, depending on the cancer type, can have either a negative or a positive effect in disease initiation and progression [98,99]. To date, it remains a *terra incognita* as to whether some radiotherapeutic modalities also trigger this phenomenon. Another hypothesis is that the axon guidance processes identified in Figure 3B reflect associations between IL-18 and neuroinflammation and neurodegeneration (which are conditions further related to high-LET irradiation [100]). IL-18 is constitutively expressed in resident cells of the central nervous system (CNS), supporting a local IL-18-dependent immune response that can influence neural tissue homeostasis [101,102]. Investigating how carbon ion beams, compared to other radiation types, may activate neuronal pathways, and how this reflects to the tissue microenvironment and the crosstalk of the irradiated cells with surrounding neuronal and immune cells, remains a subject of fruitful research. Dissecting the connection between therapeutic radiation and the co-option of neuronal programs in the irradiated cells could provide invaluable insights for increasing the therapeutic efficacy of radiation and ameliorating any side effects on healthy tissues. 

### 3.3. Radiation Type-Specific Disease Pathways Inferred from Transcriptomes of Irradiated Cells

An analogous analysis of the overrepresented human disease pathways that are associated with irradiation-responsive transcripts indicated relatively distinct disease profiles across radiation types (Figure 3C and Table S3). In detail, liver dysfunction pathways are dominant upon X-ray irradiation (Figure 3C and Table S3), perhaps as a sequalae of the critical function of this organ in the protection against induced stress, hence indicating a sensitivity of the liver upon radiotherapy. Another vital organ that might be affected is the heart since carbon ion irradiation was found to be associated with atherosclerotic disease (Figure 3C and Table S3), in agreement with clinical reports that patients who have undergone radiotherapy are at increased risk for cardiovascular diseases (CVDs) [103]. Since IL-18 participates in atherogenesis [104], the increased incidence of CVDs might reflect the activation of IL-18 signaling pathways that are associated with this radiation type. These findings suggest that increased monitoring, further investigation and timely treatment might be required in order to prevent these unwanted effects. Proton-based therapy appears to be related with inflammation and fever (Figure 3C and Table S3), two mild side effects that are amenable to clinical management. Energetic carbon and alpha particles are associated with reperfusion injury (Figure 3C and Table S3), a type of ROS-induced tissue damage occurring when blood supply returns to tissue after a period of ischemia or hypoxia. Interestingly, single-dose radiotherapy coupled with early tumor ischemia/reperfusion can lead to tumor lethality via the inactivation of homologous recombination [105]. Hence, occurrence of this side effect in patients undergoing radiotherapy might be an indicator of selective tumor radiosensitization and increased therapeutic efficiency. In conclusion, our analysis reveals radiation type-specific side effects and possible comorbidities that call for increased surveillance for relevant patient complaints after radiation treatment.

### 3.4. Machine Learning-Generated Gene Signatures of Cell Sensitivity to High- Versus Low-LET Radiation Types

**Figure 4:**
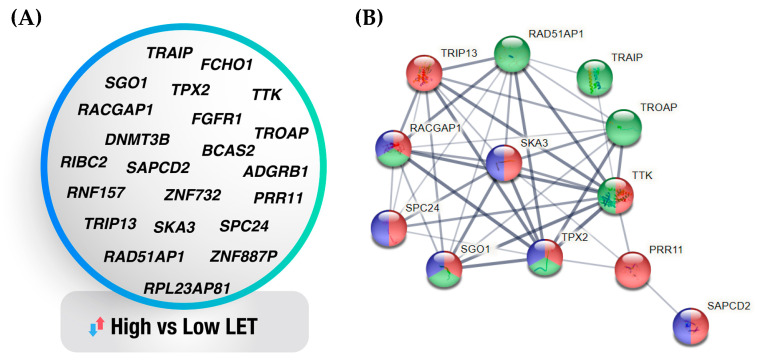
Gene signature of high- *versus* low-LET radiation types. (**A**) Twenty-two-gene signature characteristic of response to high-LET vs. low-LET irradiation. (**B**) Network depicting the associations (edges) of twelve signature genes/gene products (nodes) in cell cycle (red), cell division (blue) and inflammation (green).

One issue in the clinic is the selection of individual patients for high- or low-LET radiation treatment, which is in turn dependent on the radiobiological properties of the tumor [106]. In this regard, transcriptomics data of irradiated cells can infer radiosensitivity predictors, whereby differentially expressed genes are ranked on the basis of certain evaluation criteria, and then an optimal subset of genes is selected [107]. Although insightful, previously described methods may pose limitations in gene selection, as the produced gene signatures may not accurately capture the relationship between the selected genes and the precision of the classification. To bypass these limitations, we applied machine learning, a robust computational method that holds promise to reduce the complexity of whole genome gene expression patterns and produce manageable signatures of response while simultaneously taking into account several important selection criteria [108]. We used a recently-developed algorithm based on recursive feature elimination (RFE), which creates gene sets recursively and then determines an optimal subset, aiming to obtain the smallest possible sets of variables in an iterative way while discarding those genes of least importance [36]. To verify the ability of the algorithm to generate gene signatures linked to the features of interest, we initially ran a control test in DEGs of cancer versus normal tissues that are included in the RadBioBase. The algorithm predicted correctly a number of markers of tumor initiation and progression, such as CD44 [109], MMP9 [110], CDC20 [111], FOS [112] and WNT5A [113] (Figure S1). Several of these genes are also associated with sensitivity to radiation, as confirmed by further comparisons versus our previously published comprehensive lists of molecular determinants of radiation response in cancer tissues [114]. Having assured the accuracy of the algorithm in our datasets, we proceeded to generate a gene signature (Figure 4A) for high- *versus* low-LET radiations, using clinically relevant criteria such as post-irradiation time and dose on the data of RadBioBase. The five types of radiation were grouped into two groups because the larger the dataset, the more information the machine learning algorithm can capture, thereby enhancing its predictive performance. This led to the identification of a 22-gene signature that is characteristic for the response to high-LET as opposed to low-LET irradiation. GSEA analysis showed that the most significantly enriched (FDR < 0.05) processes of those genes are cell cycle, cell division and inflammation. 

A further STRING analysis of this signature revealed that twelve of those genes/proteins appear to interact (Figure 4B) and mediate cell cycle, cell division and/or inflammation (Figure 4B, genes with red, blue and green color-coding), thereby accurately reflecting the main processes known to be induced by LET. Among these genes, we were able to identify several recently-characterized effectors of radiosensitivity, such as RAD51-associated protein 1 (RAD51AP1), which plays an integral role in homologous recombination by activating RAD51 recombinase, and its knockout is shown to induce radiosensitivity [115]; TTK protein kinase, the inhibition of which radiosensitizes basal-like breast cancer cells through impaired homologous recombination [116]; the DNA methyltransferase 3B (DNMT3B), an epigenetic modifier that protects centromere integrity by restricting R-loop-mediated DNA damage [117], and its silencing can restore the p53/p21 signaling pathway via DNA demethylation [118]; and TRAIP, a novel RAP80-interacting protein that is necessary for translocation of RAP80 to DNA lesions and promotes homologous recombination in response to DNA damage [119]. This signature also revealed novel genes that are associated with the response to radiation, for example, the Spindle And Kinetochore Associated Complex Subunit 3 (SKA3) and the Rac GTPase Activating Protein 1 (RACGAP1). Future clinical validation of this signature in tissues from patients that have undergone high-LET radiation therapy can define indicators of responsiveness in this therapeutic modality towards improving patient selection.

### 3.5. Low-Dose Irradiation Is Associated with Cytokine Cascades, While High with ROS Metabolism

**Figure 5:**
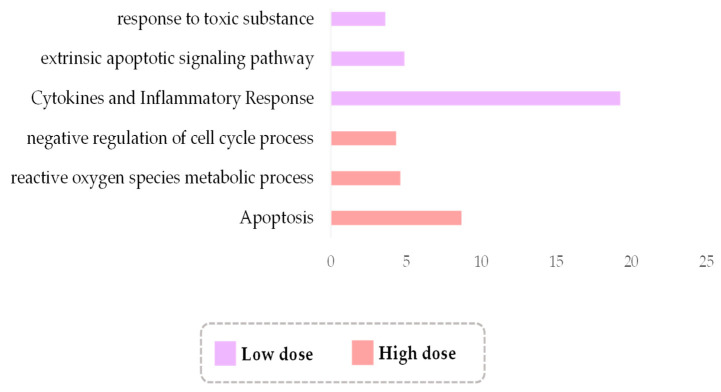
Overrepresented biological pathways (affinity propagation) for all 445 DEGs in low-dose (0.3–0.5 Gy) radiation group and all 668 DEGs in high-dose (0.6–2.0 Gy) radiation group considering all types of irradiation. The *x*-axis corresponds to the enrichment ratio.

Thus far, the implementation of new technologies in radiotherapeutic treatment has been largely empirical and driven by the belief that increasing doses will increase cure [1]. Consequently, in a large number of studies, high doses have been preferentially used to address the effects of irradiation on tissues. However, increased doses pose clinical risks for acute and/or chronic toxicities, without substantially enhancing the therapeutic benefits. Moreover, there is emerging evidence that low doses can be beneficial against several pathological entities. For example, in cancer, low-dose irradiation reverses resistance to immunotherapy by reprogramming the TME of immune-cold tumors [120], while in COVID-19-induced pneumonia, it induces antiinflammatory responses [121]. To further explore whether low doses could have therapeutic potential, we mined the RadBioBase database for differences in transcriptomes induced at different doses. The database contains entries from 37 studies using doses over 5 Gy, 2 studies using less than 0.5 Gy and 3 studies using both. For our analysis, we particularly considered the entries with a value of 0.3–0.5 Gy as “low” and those with a value of 0.6–2.0 Gy as “high” since these correspond to the clinically relevant low/high dose ranges. A GSEA analysis for the 445 genes found commonly deregulated at the 0.3–0.5 Gy range, underscored a profound overrepresentation of cytokine and inflammatory response pathways, implying that low doses are capable of inducing inflammation-related cascades. This is distinct from effects at high doses, where the 668 genes commonly responding to the 0.6–2.0 Gy range are associated with ROS metabolism (Figure 5 and Table S4). Following a gene-centric approach, we found many up or/and downregulated cytokines and interleukins, as well as other inflammation-related genes, deregulated at “low” doses. These include, but are not limited to, the upregulation of antiinflammatory genes *IL4* and *TNFA1P3*, and downregulation of the proinflammatory genes *IL12B* and *CDK5R2*. Nevertheless, genes that can exert both anti and proinflammatory activity depending on cell content, for example, *IL1A*, *IL1B*, *IL6* and *CXCL3*, appear to be upregulated at low doses in the original dataset, implying a complex cytokine profile at this range. The transcription of cytokines and other secreted molecules mediating intercellular communication (e.g., *CCL3*, *CCL4*, *CXCL2*, *IL22*, *TNF*, *IL18R1*, *IL7R* and *IL13RA2*, *IL13* and *IL10)* were also deregulated at high doses. Hence, low doses alter the transcription of secreted factors, but the composition of these factors is distinct compared to that of the high dose. In support, it was recently shown that high and low doses of irradiation induce different secretome profiles [122]. Given that our analyses are inevitably based on a relatively small number of available studies at low doses, further comprehensive characterization of the secretomes of low-dose irradiated cells is required to confirm these findings and decipher the inflammatory molecules with *bona fide* effects from those related to toxicities and radioresistance [123]. Considering that different doses/types induce different kinds of DNA damage, future high-throughput identification and functional characterization of the secretomes of cells irradiated with several types and/or doses holds promise to unveil links between intrinsic cell damage and the effects on adjacent and remote tissues, which can be translated into improved clinical patient management. 

## 4. Conclusions

The effects of irradiation are cell-intrinsic and cell-extrinsic, with the ability to reprogram the microenvironment both proximal and distal to the irradiated sites. Each irradiation type is suspected to cause different initial DNA lesions and activate distinct DDR/R components, inducing cell–cell interactions that ultimately lead to distinct immunogenic effects on cancer cells and on remote normal tissues. Predicting and characterizing the track of localized and systemic effects for each radiation type and dose can help fine-tune radiotherapy used alone or in combination with chemo- or immunotherapies, in a way that is less empirically-based and more guided by solid clinical and radiobiological data. To this end, comprehensive comparisons of changes in gene expression across normal and cancer cells for the several types and/or doses of radiation have a high clinical value for informing and improving decisions for radiotherapy. To address these novel challenges, we developed a database, termed RadBioBase, that can provide systemic insights into the attributes of irradiation relative to gene transcription in mammalian tissues. Further extending and updating this database to include additional tissue types in the future is anticipated to provide a cornerstone for the in silico prediction of the beneficial and toxic effects of radiation locally and systemically, which can be translated to more efficient and safer radiotherapy schemes. On the one hand, analyses of transcriptome changes in cancer cells can reveal novel pathways that enhance the response to radiation and/or awaken the immune system against the tumor cells. On the other hand, analyses of normal tissues can indicate genes associated with radiation type-specific side effects.

Notably, our database is designed to provide correlations between irradiation and the full-length transcripts of genes. At this point, it should be mentioned that several genes can synthesize isoforms or mutant forms with distinct or even opposing functions. Members of the TP53 family constitute such representative cases. For example, while wild-type TP53 induces radiosensitivity, expression of its missense mutants correlates with radioresistance [124]. Similarly, TP73, a sibling of TP53, synthesizes not only full-length TAp73 isoforms, which sensitize cells to irradiation, but also N-terminal truncated isoforms that are generated via aberrant splicing or alternative promoter usage at the 5’end and act as dominant negative inhibitors of their TAp73 counterparts, favoring resistance to radiation [125,126]. In cases of such genes, where their various protein products exert divergent effects on DDR and radiosensitivity, our database detects general associations with irradiation, without deciphering among the functionally divergent isoforms. The involvement of alternative forms or gain-of-function mutants needs to be subsequently addressed in a more detailed, gene-centric manner, using complementary targeted next generation sequencing approaches.

Last but not least, the COVID-19 pandemic has changed our world by accelerating new digital and virtual reality megatrends in healthcare and setting in motion a dynamic that is expected to last and reform society and science at several levels. These changes are now more than ever before extrapolated to radiotherapy, a field that has historically evolved by taking advantage of contemporary technological trends. An important lesson taught is that central databases that share and disseminate information can improve global digital healthcare at several levels [127]. In line with this trend, our initiative to collect and systemically organize all available molecular information on the responses of mammalian tissues to irradiation can become a useful means for driving radiation oncology towards a new exciting digital health era.

## Data Availability

All data and analysis methodologies are contained in the manuscript. Any additional data requests can be addressed to the corresponding author.

## Supplementary Materials

The following supporting information can be downloaded at: https://www.mdpi.com/article/10.3390/antiox11112286/s1, Figure S1: Markers of tumor initiation and progression; Table S1: MCDS simulation results for double-strand breaks (DSBs) and total clusters of DNA damage (DSBs and non-DSB oxidative lesions) per Gy per Gbp for radiation types used in this work; Table S2: Overrepresented biological pathways of the X-ray, γ-ray, protons, carbon ions and α-particle types of radiation and the corresponding retrieved genes; Table S3: Overrepresented disease pathways of the X-ray, protons, carbon ions and α-particle types of radiation and the corresponding genes; Table S4: Overrepresented pathways and related genes of the high (0.6–2.0 Gy) and low (0.3–0.5 Gy) radiation doses for all types of irradiation; Figure S1: Gene signature of cancer *versus* normal cells/tissues. Those genes implicated in radioresistance are underlined.
