# Supplementary material for: Radiation Type- and Dose-Specific Transcriptional Responses across Healthy and Diseased Mammalian Tissues
Source: Antioxidants (Basel). 2022 Nov 18;11(11):2286. doi: 10.3390/antiox11112286 (PMC9687520; doi:10.3390/antiox11112286)
Supplement: Supplementary file 1 [file antioxidants-11-02286-s001.zip › antioxidants-1895792-supplementary.pdf]

antioxidants

MDPI

Supplementary Materials

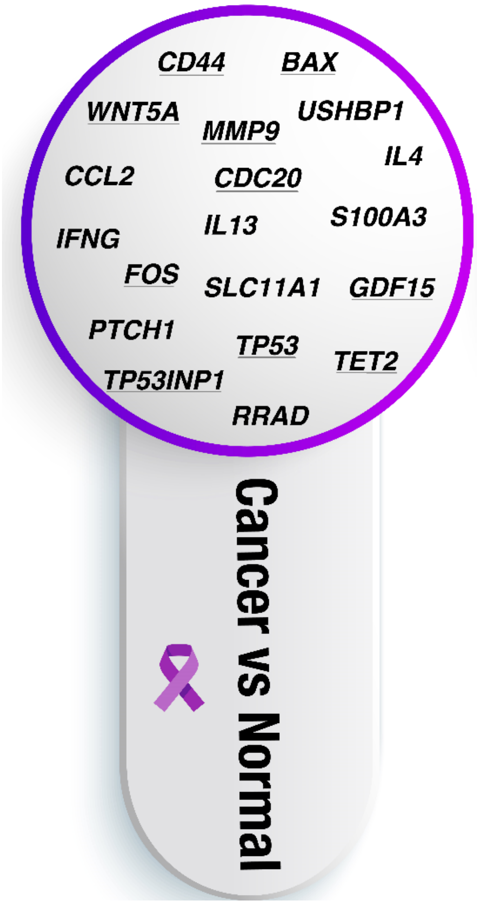

Figure S1. Markers of tumor initiation and progression.

Table S1. MCDS simulation results for double strand breaks (DSBs) and total clusters of DNA damage (DSBs and non-DSB oxidative lesions) per Gy per Gbp for radiation types used in this work.

Type of radiation	LET (Bragg Peak) (keV/μm)	Energy (MeV, MeV/n, MeV/u)	DSB (per Gy per Gbp)	All clusters (DSBs + non-DSB oxidative lesions (per Gy per Gbp)
x-rays & γ-rays	1-3	0.01 Mev (electron beam simulation)	12.780	499.7
protons	9.54*	4.5 MeV	10.290	562.1
	5.4	62 MeV	8.281	615.9
	-	70 MeV	8.259	616.3
	-	100 MeV	8.219	617.6
	-	190.6 MeV	8.168	618.9
	3.96	200 MeV	8.150	619.1
	-	210 MeV	8.156	619.2
	-	230 MeV	8.139	619.3
	-	250 MeV	8.145	619.3
Carbon ions	31.6	80 MeV/u	12.584	504.7
	33.7	75 MeV/u	12.793	499.3
	50	46 MeV/u**	14.725	453.0

	73	27 MeV/u**	17.452	390.5
	75.19	26 MeV/n**	17.686	384.6
α-particles	125	3.06 MeV**	24.765	231.2
	127.4	2.97 MeV**	24.844	228.4

*SRIM results for LET calculated at the entrance point. **Bragg Peak energy calculated from SRIM (carbon ions) or LET-Energy curves (α-particles) in accordance to the given the Bragg Peak LET.

Table S2. Over-represented biological pathways of the X-ray, γ-ray, protons, carbon ions and α-particle types of radiation and the corresponding retrieved genes.

	Gene Set	Description	Enrichment Ratio	Genes
X-rays	GO:0006631	fatty acid metabolic process	2.329	PDK4 NR4A3 AKR1C3 CD74 UCP3 ACSS1 MYO5A ADIPOR1 TH SNCA CD36 ACOT8 ACSL1 PTGES SIRT1 ACBD5 AASDH PRKAB1 PDK3 GSTZ1 PECR MGST1 PLIN5 DGAT2 GSTM2 C3 PCK1 DECR2 ACAT2 ABCD3 PHYH THNSL2 NR1H3 GCDH ACOX1 APOA5 DECR1 SREBF1 POR PON3 FADS1 ACAD11 AMACR ACAA2 MGLL ECI2 HADH ECHS1 THEM4 CPT2 PRXL2B ACADVL PDK2 SCP2 INSIG1 GSTA1 ETFB GSTM4 TRIB3 EHHADH HACD2 ABHD5 ACADL PEX7 PDP2 ACADM ACAT1 MLYCD PTGR1 NDUFAB1 HIBCH ACSF3 ACADS GPAT4 CBR4 MSMO1 ETFDH HACD3 HSD17B4 PPARA MCEE IVD PRKAA2 AIG1 PPARGC1A PTGR2 CRYL1 PLP1 CPT1A ELOVL7 PRKAR2B INSIG2 ACACA ADIPOR2 PLA2G4A TBXAS1 DAGLB LPIN2 PTGS1 ECH1 ALOX12B ALOX12 CROT PON2 MLXIPL SLC27A5 ALOX15 LPL FABP3 ALOX5 FABP5 PTGIS
	GO:0044282	small molecule catabolic process	2.175	HMMR APOBEC3H GLS2 NUDT15 AKR1C3 IL4I1 BPGM NT5C3A DPYS HAGH HEXB PDXP ACMSD GLS ACOT8 GAPDHS ACBD5 PADI4 MPST GSTZ1 PECR PRODH PLIN5 GPT UPB1 XYLB SULT1A1 LDHD PCK1 DHDH DECR2 ACAT2 ABCD3 PHYH TAT THNSL2 GCDH ACOX1 GCAT DECR1 ENTPD8 QPRT PON3 ACAD11 DCXR KYAT1 KHK PGM1 AMACR ACAA2 ECI2 HADH MCCC2 ECHS1 RIDA QDPR GOT1 CPT2 ACADVL GCSH BCKDHB SCP2 HNMT ETFB LYVE1 ALDH4A1 HYAL2 BDH1 SORD GLYCTK ALDH6A1 EHHADH NUDT1 GK ACADL PFKM OAT PEX7 GLUD1 SHMT2 ACADM ACAT1 MLYCD SDSL HIBCH ALDOC ACSF3 SCARB1 ACADS AFMID ETFDH MCCC1 HSD17B4 PPARA MCEE IVD BCKDHA HMGCL ALDH1B1 GOT2 ADH5 ENTPD5 AIG1 PCYOX1 HAAO GLUL CRYL1 NT5M CPT1A GLB1 ESD NUDT3 HSD3B7 LPIN2 TPI1 NAGK ECH1 NT5E GAPDH ALDH2 CROT APOE ALDH7A1 FAH HAL CD44
	GO:0044843	cell cycle G1/S phase transition	2.074	GLI1 CCND1 CCNA2 CDC25C CDC45 CDC6 CDK1 CDKN2C CDKN3 CDT1 GTSE1 IQGAP3 MCM10 ORC1 RRM2 AURKA BAX CDKN1A GADD45A MDM2 PCNA MYC TFDP2 PHF8 CUL4A CRLF3 PML RPA1 RGCC MCM5 FBXO7 CASP2 MCM3 MCM4 PTPN6 RBL1 FBXO5 ATM RPS6 POLE2 MTBP MCM2 CDC25A E2F8 ORC6 ID4 PRIM1 BCL2 TRIAP1 E2F7 RPS27L PLK2 POLE3 E2F1 KANK2 UBE2E2 RHOU PLK3 PCBP4 WEE1 ACVR1 PLCB1 CCNA1 FHL1 NACC2 ITGB1 CTDSPL CDKN2B BTG2
	GO:0097193	intrinsic apoptotic signaling pathway	2.032	BRCA2 DDIAS E2F2 MELK AEN BAX CDKN1A MDM2 PHLDA3 PPP1R15A TNF DDIT3 ELL3 CD74 CD24 YBX3 CUL4A HIPK1 SERINC3 PARP1 FNIP2 PML LCK CASP2 DDX5 RPS7 ATM RAD9A BMF USP28 SFPQ LRRK2 SIRT1 SKIL BCL2L1 BCL2 JMY BBC3 TNFRSF10B TRIAP1 TRIM32 RPS27L FIGNL1 HIPK2 E2F1 PRODH CIDEB PERP BNIP3 P4HB PDK2 EPHA2 TRIB3 AIFM1 FHIT BCL2L2 CLU PLEKHF1 DAB2IP PTPMT1 SOD1 SELENOS HERPUD1 PTPN1 PPIF PTTG1IP NCK2 NACC2 TMBIM6 CXCL12 CD44 NOX1 CEBPB HIC1 PYCARD HSPB1 NUPR1 CHAC1
	GO:0006520	cellular amino acid metabolic process	1.984	GLS2 IL4I1 PHGDH SERINC3 TH AZIN1 SLC7A5 DPYS SMS ACMSD MTR GLS CPOX AASDH ASNS SLC7A6 P4HA2 PADI4 MPST GSTZ1 KYAT3 PRODH GPT AGMAT UPB1 TAT PEMT THNSL2 GCDH GAMT GCAT ASL SRR KYAT1 MMUT MCCC2 RIDA DGLUCY QDPR PRDX4 GOT1 P4HB SEPHS2 ACY1 GCSH BCKDHB BPHL HNMT NIT2 ALDH4A1 ALDH6A1 FN3K OAT GLUD1 SHMT2 GATB ACAT1 SDSL HIBCH GCLC ADI1 AFMID GSS SCLY NMNAT1 MCCC1 IVD BCKDHA EGLN3 HMGCL MTHFD1 GOT2 MSRA PCYOX1 PPA1 HAAO GLUL MTHFD2L ODC1 CKB COMT ALDH7A1 FAH HAL HNF4A

	GO:0016053	organic acid biosynthetic process	1.962	PDK4 GLS2 AKR1C3 CD74 BPGM ACSS1 PHGDH SERINC3 MYO5A ADIPOR1 MGST2 OGT MTHFD1L ACMSD MTR GLS ACOT8 GAPDHS MGST3 PTGES SIRT1 ASNS PRKAB1 PECR MGST1 GPT OSBPL1A UPB1 DECR2 ABCD3 FGFR4 THNSL2 NR1H3 GAMT APOA5 CYP27A1 ASL SLC35D1 FADS1 SRR KYAT1 PGM1 AMACR MGLL ALDH8A1 GOT1 PRXL2B ACADVL SEPHS2 SCP2 INSIG1 GCH1 GSTM4 TRIB3 PFKFB1 HACD2 ACADL PFKM OAT GLUD1 SHMT2 MLYCD NDUFAB1 ALDOC ACSF3 ADI1 CBR4 HACD3 STARD4 UGP2 HSD17B4 PPARA PRKAA2 MTHFD1 GOT2 ENTPD5 PPARGC1A HAAO GLUL MTHFD2L RBP1 ABCC5 PLP1 ELOVL7 INSIG2 ACACA ADIPOR2 PLA2G4A HSD3B7 TBXAS1 LDHA TPI1 PTGS1 ALDH1A2 ALOX12B GAPDH ALOX12 MLXIPL SLC27A5 ALOX15 CLTC SIRT6 LPL ALOX5 FABP5 PTGIS
	GO:0006732	coenzyme metabolic process	1.908	PDK4 FDXR BPGM ACSS1 SNCA RFK PNPO OGT MAT2B MTHFD1L NAPRT CD38 PDXP ACMSD PANK4 ACOT8 ACSL1 GAPDHS NADK2 MTHFD2 PDK3 GSTZ1 DGAT2 SLC46A1 PEMT GCDH GAMT QPRT DCXR PGM1 MCCC2 QDPR THEM4 SHPK PDK2 H6PD SPR GCH1 DBI PFKFB1 HACD2 IDH1 MVK PFKM PDP2 SHMT2 ACAT1 MLYCD MDH1 PDXK GCLC ALDOC ACSF3 COQ10B GPAT4 AFMID MOCS2 NMNAT1 MCCC1 HSD17B4 PPARA MCEE SUCLG2 PCCB CYB5A COQ7 PRKAA2 SLC25A1 HMGCL MTHFD1 ENTPD5 PCCA GPHN MOCOS PPARGC1A HAAO MTHFD2L SLC2A3 ELOVL7 ACACA LDHA TPI1 NT5E CYB5R3 GAPDH CROT MLXIPL SIRT6 NOX1 BST1 PTGIS
	GO:0006790	sulfur compound metabolic process	1.908	PDK4 CHST3 ISCA1 ACSS1 PHGDH SNCA HSCB MGST2 ISCU HAGH TSTD1 MAT2B SMS GSTM5 HEXB MTR ACOT8 ACSL1 MGST3 PTGES GSTT1 MPST PDK3 ABHD14B PAPSS2 GSTZ1 LPO MGST1 GSTM1 DGAT2 GSTM2 SULT1A1 GSTK1 OPLAH PEMT GCDH GAMT SLC35D1 MMUT SQOR MCCC2 THEM4 PDK2 GSTT2 GSTA1 NAT8 GSTM4 SLC19A2 DBI HACD2 IDH1 MVK PDP2 ST3GAL3 ST3GAL4 ACAT1 MLYCD ARSG GCLC SLC35D2 ACSF3 ADI1 GPAT4 NUBPL GSS MCCC1 HSD17B4 MCEE SUCLG2 PCCB GSTA2 SUOX SLC25A1 HMGCL MTHFD1 SOD1 MSRA GSTA5 PCCA BPNT1 PCYOX1 MTHFD2L ELOVL7 CSGALNACT1 GLB1 ESD ACACA ANGPT1 CNDP2 COMT CHAC1
	GO:0002521	leukocyte differentiation	1.841	VEGFA CLEC4D MYB BAX TNFSF4 FOS KLF6 EGR3 TNF EGR1 MYC GPR68 CCR6 CD79B CD79A LY6D BLNK CCR7 CD74 CA2 CR2 GPR183 CD83 CD8A GPR18 IRF4 TCF7 BCL11B LEF1 RSAD2 PRXL2A CARD11 SLAMF6 IL7R DOCK10 CUL4A SATB1 FOXP1 CD3D ITK RASSF2 CD2 PARP1 MALT1 DTX1 PLCG2 CD3E SASH3 TGFBR2 RASGRP1 CCR9 LY9 MEF2C LCK CLCF1 NFATC2 FBXO7 LYL1 CAMK4 DOCK11 CMTM7 CD3G PTPN6 TCF3 CD27 ITGA4 TESPA1 RELB POU2F2 ATM TFRC RPS6 BTK ITM2A CBFB FANCA THEMIS IFNA1 GATA3 SIRT1 IKZF1 GLI3 BCL2 GAB2 TNFSF8 TNFSF9 ZFPM1 GAS6 TMEM176B C1QC RORC PIR MAFB FARP2 FAM20C TMEM176A LTBR RORA CD4 EPHA2 CEBPA IL18 TRIB1 AXL CTNNBIP1 SOD1 IL5 PRKCA TAL1 PTPRJ LGALS9 GATA2 MITF DNAJB9 TSPAN2 ITGB1 SNX10 TRPM2 CEBPB APP GAB3 SHH GLI2
	GO:0044772	mitotic cell cycle phase transition	1.738	CCND1 AURKB CCNA2 CCNB2 CDC20 CDC25C CDC45 CDC6 CDCA5 CDK1 CDKN2C CDKN3 CDT1 CENPE CENPF CIT CLSPN DTL ESPL1 FOXM1 GTSE1 HMMR IQGAP3 MAD2L1 MCM10 MELK NDC80 NEK2 ORC1 PKMYT1 PLK1 PLK4 RRM2 TPX2 TRIP13 TTK UBE2C AURKA BAX CDKN1A GADD45A MDM2 PCNA TEX14 MYC CDC25B TFDP2 PHF8 CUL4A CRLF3 MTA3 PML FZR1 RPA1 RGCC MCM5 FBXO7 CASP2 MCM3 MCM4 PTPN6 UBE2S RBL1 FBXO5 DONSON FBXL12 ATM RPS6 POLE2 MTBP MCM2 CDC25A E2F8 ORC6 ID4 PRIM1 RAD17 CEP76 FOXO4 BCL2 PPM1D TRIAP1 XPC E2F7 RPS27L PLK2 CKS1B ANLN TUBA4A POLE3 E2F1 KANK2 UBE2E2 RHOU PHLDA1 NEK6 PLK3 PCBP4 WEE1 ACVR1 PLCB1 CCNA1 FHL1 YWHAG PRKAR2B HAUS6 NACC2 ITGB1 CTDSPL EPS8 CDKN2B APP PINX1 BTG2
γ-rays	WP4286	Genotoxicity pathway	19.188	BTG2 TP53I3 AEN BLOC1S2 IKBIP PHLDA3 TRIAP1 DDB2 GADD45A CDKN1A PPM1D PRKAB1 SERTAD1 TIGAR PTGER4 TRIM22 RBM12B ACTA2
	WP3529	Zinc homeostasis	12.706	MT1A MT1E MT1F MT1G MT1H MT1X MT2A
	WP3286	Copper homeostasis	8.706	MT1A MT1E MT1F MT1G MT1H MT1X MT2A
	WP2864	Apoptosis-related network due to altered Notch3 in ovarian cancer	8.706	THBS1 HSPA5 NQO1 IER3 TNFRSF10B PTK2 CDKN1A
	WP707	DNA Damage Response	7.787	CCNB2 BAX FAS TNFRSF10B DDB2 GADD45A CDKN1A SESN1
	GO:1901654	response to ketone	4.264	KLF2 THBS1 GNRH1 PRKCE CD38 CA9 CDKN1A TNFSF4 CCR7 PTGER4 STAR NPAS4
	GO:0071241	cellular response to inorganic substance	4.177	HSPA5 NQO1 TIGAR MT1A MT1E MT1F MT1G MT1H MT1X MT2A WNK1 STAR FUS
	GO:0097305	response to alcohol	4.070	KLF2 CCR5 NQO1 GNRH1 PRKCE TP53INP1 CDKN1A TNFSF4 CCR7 PTGER4 ABCA1 LEP STAR NPAS4
	GO:0045926	negative regulation of growth	3.761	SOCS2 GDF15 DCUN1D3 PTK2 CDKN1A MT1A MT1E MT1F MT1G MT1H MT1X MT2A MINAR1 SEMA3E

	GO:0009314	response to radiation	3.184	CCL11 USP2 HSPA5 AEN BAX DCUN1D3 IKBIP TP53INP1 TRIAP1 DDB2 GADD45A POLH XPC CDKN1A PPM1D TIGAR TAF1 NF1 STAR SLC1A3 ABCA4
**protons**	WP3646	Hepatitis C and Hepatocellular Carcinoma	7.479	NFKB1 NOS2 PTGS2 PTPN11 STAT3 CXCL8 VEGFA MAPK8 FASLG CASP3 IL6 BIRC3 CASP9 CDKN1A CCND1 TP53 CD44 BRCA1
	WP254	Apoptosis	6.764	NFKB1 TNFRSF1A TRAF3 FAS TNF BCL2 BAX IKBKG APAF1 CASP8 DFFB FASLG CASP3 BNIP3L BOK CASP1 CASP4 CRADD TNFSF10 TRAF1 BIRC3 XIAP CASP9 RIPK1 BBC3 MDM2 TNFRSF25 TP53
	WP1772	Apoptosis Modulation and Signaling	6.702	NFKB1 TNFRSF1A TRAF3 FAS BCL2 MAPK8 BAX APAF1 CASP8 DFFB FASLG CASP3 BNIP3 BOK CASP1 CASP4 CRADD TNFRSF11B TNFSF10 BIRC3 XIAP CASP9 RIPK1 BBC3 TNFRSF6B TNFRSF10C BLK TNFRSF25 FOS TP53
	WP4298	Viral Acute Myocarditis	6.355	SOCS1 STAT3 IL12B IL10 TNF IL2 IFNG BCL2 BAX MMP9 EIF4G1 CASP8 DFFB CASP3 CD40LG IL6 CASP1 PYCARD CASP9 CCND1 CCR5 STAT1 DMD NOS1 PTCRA SGCA
	WP4658	Small cell lung cancer	5.512	NFKB1 TRAF3 NOS2 PTGS2 BCL2 BAX IKBKG COL4A5 APAF1 CASP8 GADD45A CASP3 TRAF1 BIRC3 CASP9 CDKN1A COL4A6 CCND1 TP53 CCNE2 ITGA2 ITGAV LAMB3 LAMC1 COL4A1 RXRA
	GO:0072593	reactive oxygen species metabolic process	3.571	NOXA1 CCS NOS2 NOX1 PTGS2 SH3PXD2A SIRPA STAT3 IL10 TNF IFNG BCL2 ADGRB1 ARF4 GADD45A DNM2 MPO PRDX4 SOD3 TPO NQO1 SOD2 BNIP3 PRDX2 RIPK1 DUOX1 EPX IL19 TIGAR CDKN1A DUOX2 SESN1 CD34 PLIN5 IL1B TP53 SLC7A2 BRCA1 FANCC LPO NOX4 HBM ITGAM NOS1
	GO:0043588	skin development	2.946	CELA2A LCE5A ERRFI1 PAX6 TNF BCL2 KRT27 KRT37 DHCR24 CASP3 CASP14 CYP26B1 ABCA12 ANXA1 EPHA2 INHBA KRT16 SPRR2G WNT5A DSG4 HOXC13 KRT25 KRT28 KRT31 KRT33A KRT33B KRT35 KRT71 KRT72 KRT73 KRT81 KRT83 KRT86 KRTAP11-1 KRTAP1-3 KRTAP1-4 KRTAP1-5 KRTAP16-1 KRTAP3-1 KRTAP3-2 KRTAP4-1 KRTAP4-2 KRTAP4-6 KRTAP4-8 KRTAP4-9 KRTAP8-1 KRTAP9-3 PRR9 TCHH EDA COL1A1 ITGA2 CDH3 ITGB4 ADAMTS2 LCE4A NF1 KRTAP19-1
	GO:0006979	response to oxidative stress	2.940	UCP3 IDH1 CCS GPX6 GPX7 MB NOX1 PTGS2 PRNP SIRPA MYB IL10 TNF BCL2 MAPK8 CCNA2 MMP9 DHCR24 GPX2 ALS2 DNM2 MPO PRDX4 SOD3 TPO TXNRD1 CASP3 DAPK1 NQO1 SOD2 TP53INP1 IL6 BNIP3 PRDX2 RIPK1 DUOX1 EPX XPA ANXA1 DUOX2 GNAO1 MDM2 SESN1 PLEKHA1 PPIF PTGS1 FOS TP53 GJB2 NR4A3 UCP2 GUCY1B1 STAT1 FANCC MGMT LPO NOX4 COL1A1 MMP14 ALDH3B1 GPR37
	GO:0008544	epidermis development	2.785	CELA2A LCE5A ERRFI1 PAX6 USH2A HES1 TNF BCL2 FGFR1 PTCH1 KRT27 KRT37 CASP3 CASP14 CYP26B1 ABCA12 ANXA1 EPHA2 INHBA KRT16 SPRR2G ZNF750 WNT5A DSG4 HOXC13 KRT25 KRT28 KRT31 KRT33A KRT33B KRT35 KRT71 KRT72 KRT73 KRT81 KRT83 KRT86 KRTAP11-1 KRTAP1-3 KRTAP1-4 KRTAP1-5 KRTAP16-1 KRTAP3-1 KRTAP3-2 KRTAP4-1 KRTAP4-2 KRTAP4-6 KRTAP4-8 KRTAP4-9 KRTAP8-1 KRTAP9-3 PRR9 TCHH EDA LAMB3 CDH3 DCT LCE4A LRTOMT NF1 KRTAP19-1
	GO:0009636	response to toxic substance	2.581	NFKB1 CCS GPX6 GPX7 MB PTGS2 AVP SIRPA MYB STAT3 IL10 TNF IL2 IL13 BCL2 BAX DNMT3B GSTK1 GPX2 CASP8 DNM2 MPO PRDX4 SOD3 TPO TXNRD1 CASP3 NQO1 SOD2 TP53INP1 IL6 BNIP3 PRDX2 RIPK1 DUOX1 EPX XPA ANXA1 CDKN1A DUOX2 GNAO1 MDM2 SESN1 PLEKHA1 PPIF PTGS1 CSF3 CCND1 FOS NR4A3 SLC7A8 TNC STAT1 FANCC MGMT LPO COL1A1 CHRNA7 CNR1 GPR37 HBM SLC30A4
**Carbon ions**	WP2431	Spinal Cord Injury	16.979	EGFR ICAM1 PRKCA VCAN MMP9 CCND1 CCL2 EGR1 CXCL2 IFNG FOS TNF PTGS2 NR4A1 MIF BTG2 GADD45A
	WP4659	Gastrin Signaling Pathway	15.770	VEGFA EGFR FYN PIK3CA PIK3R1 PRKCA ROCK1 SOS1 CCND1 EGR1 JUN KLF4 FOS PTGS2 CDKN1A
	WP4754	IL-18 signaling pathway	11.169	VEGFA BCL2 FN1 ICAM1 PIK3R1 PRKCA MMP9 CCL2 ATF3 JUN CCL3 CCL4 CXCL2 IFNG ACOD1 TNFSF11 FOS HSPB1 TNF PTGS2 CEBPB NR4A1 SOCS3 CXCL3 BTG2 FAS
	GO:0090130	tissue migration	9.741	VEGFA DPP4 MIA3 MYH9 NF1 NRP2 PIK3CA PPARD PRKCA PTPRM ROBO1 ID1 MMP9 JUN IFNG KLF4 HSPB1 PTGS2 NR4A1 TGFBR2 PRSS3 S100A2 EPHA2
	GO:0097485	neuron projection guidance	9.681	VEGFA SHH PTCH1 SMO GLI3 SPON2 BMP7 APBB2 FYN GAB2 L1CAM MYH10 NEXN NRP2 PIK3CA PIK3R1 PRKCA PTPRM ROBO1 SOS1 GLI2
	GO:0001667	ameboidal-type cell migration	9.123	VEGFA SHH SMO BMP7 ARID5B DPP4 FN1 MIA3 MYH9 NF1 NRP2 PIK3CA PPARD PRKCA PTPRM ROBO1 ID1 MMP9 JUN IFNG KLF4 HSPB1 PTGS2 NR4A1 TGFBR2 SMURF2 PRSS3 S100A2 EPHA2
	GO:0050673	epithelial cell proliferation	7.410	VEGFA SHH PTCH1 SMO GLI1 EGFR HOXA5 LAMC1 NF1 NRP2 PPARD PRKCA PTPRM ROBO1 ID1 CCND1 CCL2 JUN TNFSF11 TNF CEBPB NR4A1 EPHA2
	WP3888	VEGFA-VEGFR2 Signaling Pathway	7.115	VEGFA BCL2 FN1 FYN ICAM1 MYH9 NEXN NR4A2 NRP2 PIK3CA PIK3R1 PRKCA ROCK1 CCND1 CCL2 EGR1 JUN CCRL2 HSPA1A DNAJB9 HSPB1 DUSP5 PTGS2 NR4A1 FAS EPHA2
	GO:0061564	axon development	6.604	VEGFA SHH PTCH1 SMO GLI3 BCL2 SPON2 BMP7 APBB2 FN1 FYN GAB2 L1CAM MYH10 NEXN NR4A2 NRP2 PIK3CA PIK3R1 PRKCA PTPRM ROBO1 SOS1 TOP2B GLI2 JUN KLF4
	GO:0002521	leukocyte differentiation	6.524	VEGFA SHH GLI3 BCL2 GAB2 HOXA7 MYH9 NF1 PIK3R1 PRKCA SOS1 GLI2 MMP9 LIF EGR1 JUN CCL3 CLEC4E IFNG JUNB TNFSF11 DNAJB9 FOS TNF CEBPB TGFBR2 EPHA2

α-particle	WP3617	Photodynamic therapy-induced NF-kB survival signaling	11.898	CXCL8 VEGFA MMP3 PTGS2 IL6 BCL2A1 CXCL2 IL1B IL1A MMP1 BIRC5 BIRC3
	WP4286	Genotoxicity pathway	8.813	TRIM22 ACTA2 GADD45A AEN DDB2 FBXO22 PHLDA3 PPM1D CDKN1A DCP1B MDM2 PRKAB1 TRIAP1 E2F7 TIGAR TP53I3
	WP2446	Retinoblastoma Gene in Cancer	7.492	TOP2A TTK HMGB2 CCNB2 ANLN CCNB1 CDC45 MCM4 RRM1 RRM2 KIF4A MCM6 POLE2 CCNA2 MCM7 SAP30 PCNA CDKN1A MDM2
	WP179	Cell Cycle	5.689	TGFB3 PTTG1 CDC20 TTK CCNB2 CCNB1 CDC45 CDC6 MCM2 MCM4 PLK1 MCM6 GADD45G GADD45A BUB1 CCNA2 MCM7 PCNA CDKN1A MDM2
	GO:0044843	cell cycle G1/S phase transition	3.793	CTDSP2 AURKA CCNB1 CDC45 CDC6 CDKN3 MCM2 MCM4 RRM2 GTSE1 MCM6 POLE2 GADD45A FAM83D CCNA2 MCM7 FBXO5 PAGR1 BAX PCNA RPS27L CDKN1A MDM2 TRIAP1 E2F7 INHBA TCIM
	WP4754	IL-18 signaling pathway	3.607	ACTA2 CDK5R2 ANP32A CA11 EEF2 KCNH2 LCK CCNB2 SPP1 CXCL8 RGS16 VEGFA NCF1 MMP3 PTGS2 IL6 CXCL3 TNFAIP3 CXCL2 BMP2 IL1B MMP1 ATF3 CCNA2 BAX BIRC3 HMOX1 IL18R1 KITLG
	GO:0007059	chromosome segregation	3.448	MEIOB NEK2 NUSAP1 TOP2A PTTG1 CEP55 CDC20 TTK PRC1 TRIP13 AURKB CCNB1 CDC6 KIF22 RMI2 SPC25 CDCA8 MKI67 PLK1 KIF4A BUB1 FAM83D DLGAP5 PSRC1 FBXO5 BIRC5 NCAPG KIF23 HJURP TENT4A GEM
	GO:0048285	organelle fission	3.402	MEIOB BMP4 LRP5 NEK2 NUSAP1 TOP2A UBE2C PTTG1 AURKA CEP55 ASPM CDC20 TTK KIF20B KIF11 CCNB2 PRC1 ANLN CKS2 TRIP13 AURKB CCNB1 CDC6 KIF22 MND1 TPX2 CDCA8 MKI67 PLK1 KIF4A LIF IL1B IL1A BUB1 DLGAP5 CCNA2 PSRC1 FBXO5 NCAPG KIF23 CCNF BORA CCNG1 TENT4A HSPA1A
	GO:0044772	mitotic cell cycle phase transition	3.207	CTDSP2 FOXO4 NEK2 UBE2C HMMR AURKA CDC20 TTK CCNB2 ANLN CKS2 TRIP13 CIT AURKB CCNB1 CDC45 CDC6 CDKN3 FOXM1 MCM2 MCM4 RRM2 TPX2 PLK1 DTL GTSE1 MCM6 POLE2 GADD45A BUB1 DLGAP5 CCNA2 MCM7 FBXO5 BORA BAX PCNA PPM1D RPS27L XPC CDKN1A MDM2 TRIAP1 E2F7 INHBA
	GO:0045787	positive regulation of cell cycle	3.155	FOXO4 LRP5 NUSAP1 UBE2C AURKA KIF20B CCNB2 CKS2 CIT AURKB CCNB1 CDC45 CDC6 DTL GTSE1 IL1B IL1A GADD45A FAM83D DLGAP5 CCNA2 PSRC1 FBXO5 KIF23 PAGR1 CCNF BAX CCNG1 PCNA CDKN1A MDM2 TRIAP1 TRIM32 E2F7

Table S3. Over-represented disease pathways of the X-ray, protons, carbon ions and α-particle types of radiation and the corresponding genes.

	Gene Set	Description	Enrichment Ratio	Genes
X-rays	C0151766	Liver function tests abnormal finding	2.392	CD19 MS4A1 CR2 POLD1 TNFRSF13B NFKB2 ATM ABCD3 SLC25A13 ACOX1 BCS1L ADK AMACR ABCB4 CPT2 SLC37A4 AGL MVK ACADM SLC25A20 SAR1B HSD17B4 F5 CPT1A FAH TFR2 HNF4A
	C0027051	Myocardial Infarction	2.301	ACTA2 BAX TNFSF4 TNF DDIT3 LTA KLK1 ATM ACE BCL2L1 BCL2 MIA3 HP CYP27A1 TM6SF2 GCLC DAB2IP LDLR SOD1 PPARGC1A MPL F5 GSK3B LMNA HSPA5 LDHA AGT ITGB3 CKB P2RY12 TIMP2 APOE PAPPA LPL
	C0086565	Liver Dysfunction	2.247	CD19 MS4A1 CR2 POLD1 TNFRSF13B NFKB2 ATM ABCD3 SLC25A13 ACOX1 BCS1L ADK AMACR ABCB4 CPT2 SLC37A4 AGL MVK ACADM SLC25A20 SAR1B PEX26 HSD17B4 PEX16 F5 CPT1A FAH TFR2 HNF4A MTM1 DDOST
	C0009375	Colonic Neoplasms	2.173	CCND1 VEGFA BIRC5 MKI67 TCF19 ACTA2 BAX CDKN1A MDM2 PCNA TNF MYC LEF1 PTP4A3 MUTYH TGFBR2 SH3KBP1 DNMT1 GSTM5 PCP4 S100A4 MCM2 TAGLN BCL2 SULT1A1 LRG1 SPARC ITGA7 PLCB1 ALDH1B1 F5 CP ID3 GUCY1A1 OXT ODC1 NDRG2 ITIH4 NOX1 LPL ALOX5 HSPB1 NOTCH1
	C3495559	Juvenile arthritis	2.097	NR4A2 GADD45A FOS FOSB HSPA6 NR4A3 DDIT3 EGR1 HBEGF DUSP2 CD83 CD8B FCHSD2 FOXP1 ADCY7 BTG3 PROS1 NEAT1 PLK2 STAB1 FAM20A CTTN RBM47 CLU AQP9 MIR22HG ITGB5 SLC2A3 DAPK1 GMPR PACSIN2 SH3BGRL2 CD14 CALD1 GP1BB ALOX12 ITGA2B SLC11A1
	C2239176	Liver carcinoma	1.941	GLI1 CCND1 BIRC5 CDKN3 FOXM1 RRM2 SPIB GDF15 FOS TNF MYC CXCL8 CA2 POU2AF1 TH PRDX2 ATM IRF5 RPS6 ARID1A SREBF2 IFNA1 ACE BCL2L1 UROD E2F1 GSTM1 USP2 PCK1 MT2A MT1A SLC25A13 ACOX1 SREBF1 EPHX1 ABCB4 THEM4 CEBPA SLC37A4 IDH1 MVK HTATIP2 PRDX6 ANXA4 MTUS1 FDFT1 CCNA1 GLUL CP PDIA3 ACACA HSPA5 TAGLN2 GAPDH COMT CTSD FAH CXCL12 MLXIPL CEBPB PYCARD FABP5 HSPB1 ADD1 SHH BTG2
	C0019209	Hepatomegaly	1.877	FOS FASLG CD19 CA2 CR2 UCP2 IL7R POLD1 IL2RG ITK TNFRSF13B KCNN4 CD96 MCM4 CD27 RASA2 SH2D1A CPOX TRIM37 KLF1 ABCD3 NR1I3 GCDH ACOX1 ASL AMACR SC5D MMUT ABCB4 CPT2 ACADVL GNE ETFB SLC37A4 ABHD5 AGL MVK NPC1 SEC63 ACADM SLC25A20 PEX26 STEAP3 SLC20A2 ETFDH HSD17B4 PEX14 PPARA PCCB PEX16 HMGCL PCCA MPL F5 CPT1A SLC39A4 LMNA GLB1 GAA LPIN2 BSCL2 SCARB2 NAGLU SNX10 APOE FAH HNF4A LYST

	C0023893	Liver Cirrhosis, Experimental	1.759	VEGFA CCNA2 CCNB2 CDK1 CDKN3 HELLS MKI67 SHCBP1 ACTA2 AEN CCNG1 CDKN1A FHL2 FXYD2 GLS2 FOS KLF6 ATF3 TNF MYC AKR1C3 GPR68 GZMA HOPX LTB CD74 TRIM59 CD83 LMO2 CD24 VOPP1 PRKCB IL7R DOCK10 LY86 IRF8 MYO5A SLC7A1 IL2RG CD36 STK10 TMSB10 S1PR1 FBXO9 SASH3 TGFBR2 MYO1C YPEL3 LBH PLD4 DPYS HAGH PTPRO PAQR9 CD48 RBM3 CD38 SMC2 NFKBIE EZR GLS ADD3 CAPG ID4 TSPAN8 TRAK2 BMF ACSL1 IFNA1 PLAC8 PRNP CTSE SIRT1 ASNS RNASEL EMP1 PRODH KRT18 GPT GSTM1 LCN2 RNASE4 PCK1 MT2A MT1A C1QA PLEKHB1 C1QC SLC46A1 SLC25A13 GCAT DIO1 SPARC SREBF1 INHBE DCXR SRR FABP7 AMACR C8G SDR42E1 IL18BP SLC10A1 NEK6 DHRS3 GOT1 NQO2 PRLR GJA4 CPQ NAT8 SLC22A23 SLC19A2 BDH1 SLC37A4 TRIB3 GK AGL TMBIM1 OAT IL18 ACAT1 MDH1 ANXA4 PLSCR1 MAP1LC3A PAMR1 IVD PLPP2 GSTA2 AXL SELENOP ALDH1B1 FAHD1 MGMT SOCS2 GRN FHL1 YWHAG ABCC5 PTPN1 CSGALNACT1 AP1S2 INSIG2 HPSE RTN4 PLA2G4A THYN1 PTGS1 NAGK TAGLN2 GCHFR CFP CMTM3 YWHAZ AGT ITGB3 PLTP ADGRE1 PDGFA NT5E NSMF LHFPL6 ITGB1 SNAP23 TSC22D1 PLIN2 TIMP2 ALDH2 CROT ITGA2B CXCL12 NDRG2 CD5L HAL KNG1 C5AR1 MVP LPL LY96 RAB32 PYCARD FCGR3A SLAMF9 ICAM2 APP TUBB6 AHNAK
	C0033578	Prostatic Neoplasms	1.517	CCND1 VEGFA BRCA2 TOP2A AURKA BAX CDKN1A GADD45A GDF15 MDM2 KLF6 ATF3 EGR1 MYC AKR1C3 FASLG CXCL8 UCP3 PHGDH SERINC3 SLC7A1 PARP1 TGFBR2 RFK IL16 HNRNPH1 MED12 DNMT1 PRDX2 ATM EZR DCAF6 DNMT3B ACE PRNP CNN3 SIRT1 BCL2 LAMC1 GSTT1 RNASEL EMP1 PDZK1 IL17RC GSTM1 IGSF5 SULT1A1 RNASE4 MT2A GSTK1 DHDH FGFR4 GRB7 DCXR AMACR BNIP3 EPHX1 ALAD P4HB GSTA1 PGRMC1 SLC31A1 LIFR FHIT CLU TOM1L1 IL18 EI24 DAB2IP ANXA4 ADI1 ZFHX3 TCN2 RXRA PPARA SELENOP EFEMP2 SIL1 CRYL1 MSR1 PDIA3 RPN2 GSK3B SELENOS ID3 HERPUD1 RAD23B SLC39A1 THYN1 SDF2L1 TBXAS1 NAGK ALDH1A2 NFIC ITGB3 NAGLU CLIC4 COMT CTSD CXCL12 LPL CTBP2 SERPINB10 MAP3K7
	C4020899	Autosomal recessive predisposition	1.346	BRCA2 CDC6 CENPE CENPF NEK2 ORC1 PLK4 TRAIP DDB2 PCNA TAC3 EGR2 SOST CHST3 CD79B CD19 CD79A SLC4A1 MS4A1 BLNK SPTA1 CA2 CR2 CIITA EPB42 CD40 CD8A WHRN BPGM IL7R PHGDH TSPYL1 IRF8 NT5C3A MYO5A GLRX5 FECH CD3D RIPOR2 ITK TNFRSF13B TH CD36 MALT1 MUTYH CD3E CD96 ARL2BP LCK ISCU CLCF1 TRAF3IP2 FBXO7 RPSA DPYS IGHMBP2 PNPO IL21R PTPRO CD3G MCM4 CD27 SAMHD1 RYR1 IL10RA FOXO1 SETX ATM SP110 MTR SLC12A6 BBS9 ORC6 CORO1A SEC23A DNMT3B ACE ASNS MPZ CCDC88A PROS1 SLC25A3 TNXB TNFRSF10B TRIM32 XPC POLH HSPG2 LAMC3 GPSM2 TRIM37 ST3GAL5 POC1A PAPSS2 KANK2 PRODH F7 UPB1 NGF GJB2 C3 PCK1 C1QA ABCD3 PHYH C1QC TAT F10 OPLAH SLC25A13 GCDH ACOX1 PRG4 GAMT CFH BCS1L FAM20A CYP27A1 ASL SPARC POR ADK SLC35D1 CLCN2 AMACR SC5D HADH EPHX1 MMUT FGFR3 AK2 UQCRQ ECHS1 ALAD ABCB4 QDPR CPT2 ACADVL ACY1 GCSH PMP22 BCKDHB GNE H6PD ETFB SPR ALDH4A1 GCH1 SLC19A2 SLC37A4 ALDH6A1 GGCX ABHD5 LIFR ACP2 AGL ITGA7 MVK PFKM HSPD1 OAT PEX7 NPC1 SEC24D ACADM XDH SLC25A20 ACAT1 MLYCD EXT1 WDPCP CFL2 SAR1B HIBCH GCLC PEX26 LTBP2 GBE1 ACADS NR5A1 MOCS2 GSS DHCR24 POMT2 NDUFV1 ETFDH SLC35A3 TCN2 HSD17B4 PEX14 IVD PCCB BCKDHA DBT CYB5A PEX16 PLCB1 SUOX AXL SLC25A1 HMGCL SOD1 LMAN1 EFEMP2 PCCA GPHN NPR2 ECM1 GLUL MPL ADGRG1 DYM GFI1B GRN F5 SLC22A18 CP CPT1A SLC39A4 PPIB KIF1C GMPPB RASGRP2 LMNA RIN2 MKKS GLB1 GP9 GUCY1A1 ACACA DPAGT1 GAA TBXAS1 LDHA TPM2 LRPAP1 TPI1 BSCL2 MYO7A SCARB2 AGT ITGB3 GP1BA NT5E ALOX12B CYB5R3 CD151 NAGLU GP1BB SNX10 BMP1 ITGA2B APOE CTSD ALDH7A1 FGB FAH FLNB PROC SERPINC1 TFR2 HYDIN LYST EPS8 LPL DDOST FCGR3A EDNRB NEB HERC2 CFTR
**protons**	C0023283	Leishmaniasis, Cutaneous	17.684	IL10 TNF CCL2 CXCL8 IFNG IL1B IL4
	C0023290	Leishmaniasis, Visceral	12.992	IL10 TNF CXCL8 CSF2 IFNG IL10RA IL6 IL1B SLC11A1
	C0993582	Arthritis, Experimental	7.074	STAT3 IL10 TNF IL17A IFNG IL13 VEGFA MPO IL6 TNFRSF11B IL1B IL4 STAT1 RAG2
	C0015967	Fever	5.092	TNFRSF1A FAS PTGS2 AVP DST PRNP STAT3 IL12B IL10 TNF IL2 CXCL8 IFNG BCL2 HLA-DPB1 SOD2 IL6 BIRC3 XPA STX11 CSF3 IL1B CCND1 STAT4 EDA SLC11A1 RAG2 COL1A1 CACNA1A MEFV NOS1 XPC
	C0035126	Reperfusion Injury	4.993	NOS2 PTGS2 SOCS1 STAT3 IL10 TNF CCL2 CXCL8 BCL2 MAPK8 MMP9 CASP8 MPO CASP3 SOD2 IL6 CDKN1A INPP5D PTGS1 IL1B FOS
	C0007786	Brain Ischemia	4.833	NFKB1 TNFRSF1A NOS2 PTGS2 STAT3 TNF CCL2 IL17A MMP9 HLA-DPB1 MPO CASP3 SOD2 IL6 CASP9 BBC3 IL1B CEBPB CCR4 TP53 UCP2 ELN
	C0032285	Pneumonia	4.542	NFKB1 TNFRSF1A TNFSF12 TNF CCL2 IL17A CSF2 IFNG IL13 DNMT3B CASP8 CD40LG IL6 CASP1 IL1B IL4 CXCR3 RAG2 CX3CL1 JAK3
	C0021368	Inflammation	4.255	NOS2 PTGS2 STAT3 IL10 TNF CCL2 CXCL8 IL17A CSF2 IFNG IL13 VEGFA MMP9 MPO IL6 CASP1 TNFRSF11B TLR9 TNFSF15 IL1B CXCR3 TBX21 TIMP1 ASIC2
	C0011853	Diabetes Mellitus, Experimental	4.155	TNFRSF1A FAS NOS2 PTGS2 PAX6 TNF IFNG BCL2 VEGFA BAX MMP9 MPO CASP3 NQO1 SOD2 IL6 IL1B TP53 UCP2 HK1 TIMP1 CX3CL1

	C0003873	Rheumatoid Arthritis	3.271	PTGS2 ABCC4 IL10 TNF CXCL8 CSF2 IFNG VEGFA HLA-DPB1 FASLG MPO SOD2 IL6 TNFRSF11B TRAF1 BLK IL1B CD40 STAT4 NR4A3 STAT1 SLC11A1 SLC25A12 HAPLN1 ACAN B3GNT9 FCGR2A RGMB
Carbon ions	C0740392	Infarction, Middle Cerebral Artery	20.052	ICAM1 MMP9 CCL2 ATF3 TNFAIP6 CXCL2 JUNB FOS
	C0004153	Atherosclerosis	14.019	VEGFA ICAM1 HSPA1B CCL2 CCL3 CCL4 IFNG TNF PTGS2 SOCS3
	C0035126	Reperfusion Injury	13.623	BCL2 ICAM1 MMP9 CCL2 EGR1 JUN CCL3 CXCL2 HSPA1A FOS HSPB1 TNF PTGS2 CDKN1A
	C0038220	Status Epilepticus	9.965	VEGFA CCL2 JUN CCL3 JUNB JUND FOS HSPB1 TNF PTGS2
	C3495559	Juvenile arthritis	8.908	NR4A2 PROS1 NEAT1 FOSB EGR1 TNFAIP6 JUN CD14 KLF4 OSM FOS DUSP1 DUSP2 GADD45A
	C0021368	Inflammation	8.707	VEGFA ICAM1 MMP9 CCL2 EGR1 CCL3 CCL4 CXCL2 IFNG TNF PTGS2 MIF
	C0009375	Colonic Neoplasms	8.155	VEGFA BCL2 APC EGFR ICAM1 MMP9 CCND1 JUN IFNG HSPB1 TNF PTGS2 TGFBR2 CDKN1A
	C0151744	Myocardial Ischemia	7.739	VEGFA ICAM1 ID1 CCND1 CCL2 CCL3 CCL4 CXCL2 HSPA1A JUNB KLF4 DUSP1 TNF NR4A1 SOCS3 GDF15
	C2239176	Liver carcinoma	5.763	SHH GLI1 APC EGFR PIK3CA MMP9 CCND1 JUN CCL3 FOS HSPB1 TNF PTGS2 CEBPB SOCS3 BTG2 GDF15
	C0020538	Hypertensive disease	5.204	BCL2 GNRH1 BBS1 CD2AP EGFR FN1 ICAM1 MYH9 NF1 VHL MMP9 CCL2 JUN CXCL2 FOS DUSP5 TNF PTGS2 TGFBR2
α-particle	C0022116	Ischemia	10.182	ACTA2 CXCL12 PPARG IL6 BAX PCNA HMOX1 SOD2 EGR1
	C0035126	Reperfusion Injury	5.963	INPP5D PPARG VIM CXCL8 PTGS2 IL6 CXCL2 IL1B IL1A CDKN1A FGF2 HMOX1 HSPA1A SOD2 PLAT EGR1
	C0009375	Colonic Neoplasms	5.131	ACTA2 FZD2 KCNH2 MAP2K6 MTHFR RHOJ ACTG2 MCM2 MKI67 S100A9 PPARG VEGFA PTGS2 IL1B CXCL10 BIRC5 SPARC BAX PCNA CDKN1A MDM2 CRYAB SOD2
	C1956346	Coronary Artery Disease	4.873	LDLR ACTA2 CXCL12 MYH11 PDGFD TGFB3 VEGFA MMP3 PTGS2 GDF15 HMOX1 ANGPTL4
	C0018800	Cardiomegaly	4.407	GAS6 SLC22A5 ACTA2 MYH11 JUP TGFB3 LBR CYP1B1 LIF IL1B FHL2 FGF2 HMOX1 MT2A RRAD SOD2
	C0011853	Diabetes Mellitus, Experimental	4.145	SREBF1 ACSL1 PPARG VEGFA NCF1 PTGS2 IL6 IL1B ATF3 BAX HMOX1 HSD11B1 SOD2 PDK4
	C0007131	Non-Small Cell Lung Carcinoma	3.995	KIT DAPK1 MXRA5 NNAT FOXM1 RRM1 CXCL8 VEGFA MMP1 ATF3 BIRC5 MDM2 RRAD SOD2
	C0151744	Myocardial Ischemia	3.520	ATP1A1 GHR PFKFB3 VEGFA IL6 CXCL2 IL1B IL1A GDF15 CXCL10 SPARC FGF2 GCH1 HMOX1 HSPA1A SOD2 PDK4 EGR2 KITLG
	C1458155	Mammary Neoplasms	2.970	KIT BMP4 DEPP1 ACTA2 AKT2 CXCL12 GDF10 EEF2 MTHFR TOP2A UBE2C HMMR AURKA PRC1 FOXM1 MKI67 SPP1 CYP1B1 VIM CXCL8 SERPINB2 MMP3 PTGS2 IL6 CXCL3 BCL2A1 CXCL2 BMP2 IL1B MMP1 BIRC5 NRG1 DLL1 BAX FASN PPM1D FHL2 MDM2 HMOX1 RRAD SOD2 ANGPTL4
	C0023893	Liver Cirrhosis, Experimental	2.437	SREBF1 INPP5D ISG20 ACTA2 CXCL12 SHISA3 S1PR1 AHNAK GHR INPPL1 OLFML2B OLFML3 RASSF4 TGFB3 YPEL3 CCNB2 CDKN3 MKI67 TRIB3 S100A9 PLIN2 ACSL1 VIM VEGFA AP1S2 LPXN PTGS2 IL33 KYNU IL1B MMP1 SLC19A2 CXCL10 ATF3 CCNA2 NRG1 SPARC IL7R AEN CCNG1 GLS2 CDKN1A DRAM1 FHL2 PRNP GPR68 G0S2 BIRC3 FGF2 MT1A CRYAB HMOX1 MT2A TSC22D1 PLAT RGS5 IGFBP2 MVP DEPDC7

Table S4. Over-represented pathways and related genes of the high (0.6-2.0 Gy) and 'low' (0.3-0.5 Gy) radiation doses for all types of irradiation.

	Gene Set	Description	Enrichment Ratio	Genes
High Doses	WP707	DNA Damage Response	8.973	CCND1 BAX BBC3 CDKN1A DDB2 GADD45A MDM2 SESN1 TNFRSF10B CDC25A RAD51 APAF1 CASP3 CASP8 FAS CASP9 CDC25C TP53 BRCA1 CHEK1 RAD17 PMAIP1 CCNB2 CCNB1
	WP254	Apoptosis	8.699	BAX BBC3 MDM2 TNFRSF10B HRK BCL2 APAF1 CASP3 CASP8 TRAF3 FASLG BNIP3L CASP1 CASP4 FAS TNFSF10 TRAF1 BIRC3 XIAP BOK CASP9 CRADD RIPK1 TNF TP53 JUN PMAIP1 PIK3R1 BIRC5
	WP1772	Apoptosis Modulation and Signaling	7.489	BAX BBC3 TNFRSF10B TNFRSF10D HRK BCL2 APAF1 CASP3 CASP8 TRAF3 FASLG CASP1 CASP4 FAS TNFSF10 BIRC3 XIAP BOK CASP9 CRADD RIPK1 FOS TP53 JUN HSPA1A PMAIP1 BIRC5

	WP4754	IL-18 signaling pathway	5.548	VEGFA ACTA2 BAX CCNA2 CD36 MAP3K7 MAPK9 PLA2G7 BCL2 FN1 BTG2 ALS2 CASP3 CASP8 FASLG FAS TRAF1 BIRC3 NOS2 NOX1 PTGS2 TNF IL12B IL10 CCL2 CXCL8 IL6 IFNG IL1B IL13 FOS MMP9 TP53 TRPC2 COL1A1 MMP14 TIMP1 TIMP3 ATF3 JUN CCL3 CCL4 CXCL2 ACOD1 TNFSF11 HSPB1 CEBPB NR4A1 SOCS3 CXCL3 ICAM1 PIK3R1 PRKCA SPP1 RGS16 NCF1 CCNB2 BMP2 IL18R1 KITLG
	GO:0045930	negative regulation of mitotic cell cycle	4.736	CCND1 TOP2A AURKA PTTG1 BAX CDKN1A GADD45A MDM2 PCNA TRIAP1 XPC E2F7 RPS27L CDC6 E2F8 FBXO5 ZWINT CDT1 E4F1 PINX1 BCL2 TOP2B BTG2 CRADD TNF IL10 CCL2 CDC20 CDC25C TP53 BRCA1 CHEK1 RAD17 KLF4 DUSP1 BMP7 APC EGFR PLK3 BUB1 AURKB CCNB1 BTG3 TTK TRIP13 PLK1 GTSE1
	GO:0072593	reactive oxygen species metabolic process	4.635	PDK4 CDKN1A GADD45A GLS2 SESN1 CD36 BCL2 DNM2 MPO PRDX4 SOD3 TPO RIPK1 CCS DUOX1 EPX IL19 NOS2 NOX1 PTGS2 TNF IL10 IFNG IL1B TP53 BRCA1 FANCC LPO NOX4 ACOD1 KLF4 PMAIP1 TGFBR2 BMP7 EGFR FYN ICAM1 SESN2 DDIT4 CYP1B1 ITGAM NCF1 HBG2 HBG1 TIGAR FOXM1
	GO:0010948	negative regulation of cell cycle process	4.361	CCND1 NEK2 AURKA PTTG1 BAX CDKN1A GADD45A MDM2 PCNA TRIAP1 E2F7 RPS27L RRM2 CDC6 DTL E2F8 FBXO5 CDT1 RAD51 FZR1 PINX1 BCL2 BTG2 APAF1 CRADD CCL2 CDC20 CDC25C TP53 BRCA1 CHEK1 RAD17 LIF KLF4 DUSP1 BMP7 MIF APC PLK3 BUB1 AURKB CCNF CCNB1 TTK TRIP13 PLK1 GTSE1
	GO:0044772	mitotic cell cycle phase transition	3.549	CCND1 NEK2 HMMR UBE2C AURKA BAX CDKN1A GADD45A MDM2 PCNA PPM1D TRIAP1 XPC E2F7 RPS27L RRM2 CDC45 CDC6 DTL CDC25A E2F8 CDCA5 CCNA2 MCM10 FBXO5 MCM4 ORC6 CDT1 ID4 FZR1 PINX1 BCL2 BTG2 DNM2 CRADD CCL2 CDC20 CDC25C TP53 BRCA1 RAD17 RAD51B KLF4 DUSP1 APC NDE1 PLK3 CCNB2 BUB1 AURKB DLGAP5 MCM6 MCM7 ANLN CDKN3 BORA CCNB1 TTK CKS2 TRIP13 CIT MCM2 PLK1 TPX2 FOXM1 GTSE1 POLE2
	GO:0048285	organelle fission	3.428	CCND1 NEK2 NUSAP1 TOP2A UBE2C CEP55 AURKA PTTG1 CCNG1 CDC6 CDCA5 CCNA2 FBXO5 ZWINT CDT1 MTFR2 KIF23 RAD51 UBE2B FZR1 PINX1 TOP2B IL1B CDC20 CDC25C WNT5A BRCA2 CHEK1 RAD51B LIF HSPA1A DUSP1 BMP7 APC NDE1 MEIOB CCNB2 BUB1 AURKB MKI67 DLGAP5 PSRC1 NCAPG ANLN CCNF BORA ASPM CCNB1 TENT4A TTK KIF20B KIF11 PRC1 CKS2 TRIP13 CDCA8 PLK1 TPX2 KIF22 KIF4A MND1
	GO:0009636	response to toxic substance	3.257	CCND1 NR4A2 BAX CDKN1A MDM2 PCNA SESN1 CD36 LDHA MT2A HAMP BCL2 TP53INP1 DNM2 GPX2 MPO PRDX4 SOD3 TPO TXNRD1 CASP3 CASP8 RIPK1 CCS DUOX1 EPX GPX6 GPX7 MB PTGS2 XPA TNF IL10 IL2 CSF3 IL6 IL13 FOS FANCC MGMT GSTK1 LPO COL1A1 TNC EGR1 JUN GNRH1 CCL3 CD14 KLF4 DUSP1 BMP7 FYN ICAM1 SESN2 S100A9 S100A8 CYP1B1 ALOX5AP SPARC CCNB1 HBG2 HBG1
**Low Doses**	WP530	Cytokines and Inflammatory Response	19.283	IL12B IL10 IL7 TNF CSF2 IL13 IL6 IL1B IFNG CSF3 IL4 CXCL2 IL1A
	WP3617	Photodynamic therapy-induced NF-kB survival signaling	16.529	VEGFA TNF CSF2 CCND1 IL6 CXCL8 IL1B MMP9 MMP3 PTGS2 BCL2A1 CXCL2 IL1A MMP1 BIRC3
	WP3624	Lung fibrosis	11.019	IL12B TNF CCL2 CSF2 IL13 CCL11 IL6 CXCL8 IL1B CSF3 IL4 MMP9 SPP1 CXCL2 FGF2 HMOX1 MT2A SMAD7
	WP2328	Allograft Rejection	7.367	VEGFA IL12B IL10 TNF IL13 CXCL12 C4B CASP8 FAS FASLG CXCL8 IL1B IFNG IL4 VIM IL1A GDNF
	GO:0033002	muscle cell proliferation	5.721	GLI1 IL12B IL10 TNF IL13 SHH BMP4 S1PR1 ELANE PDE1A PDGFD TENM4 TGFB3 CNN1 CCNB1 IL6 IFNG MMP9 PPARG PTGS2 TNFAIP3 CDKN1A MDM2 FGF2 HMOX1 SOD2 ITGA2
	WP4754	IL-18 signaling pathway	5.391	VEGFA KLF2 IL12B IL10 TNF CCL2 IL13 ACTA2 CDK5R2 ANP32A CA11 EEF2 KCNH2 LCK CCNB2 CASP8 FAS FASLG IL6 CXCL8 IL1B IFNG MMP9 BCL2 SPP1 RGS16 NCF1 MMP3 PTGS2 CXCL3 TNFAIP3 CXCL2 BMP2 MMP1 ATF3 BAX BIRC3 HMOX1 IL18R1
	GO:0097191	extrinsic apoptotic signaling pathway	4.909	IL7 TNF CSF2 BMP4 FAIM2 MAP2K5 HMGB2 CD70 CASP8 FAS FASLG IL1B IFNG BCL2 BCL2A1 IL33 TNFAIP3 IL1A ATF3 BAX TNFRSF10B GDNF G0S2 HMOX1 HSPA1A INHBA TNFRSF10C SCG2
	GO:0045926	negative regulation of growth	4.628	IL12B IL10 TNF PTCH1 BMP4 ACVRL1 ELANE FRZB G6PD SEMA4C SEMA4D GDF15 BCL2 SPP1 SERPINE2 PPARG TMPRSS4 MT1X CDKN1A MT1M MT1E MT1G MT1H MT1B MT1A CRYAB HSPA1A INHBA MT1F MT2A
	GO:0009636	response to toxic substance	3.633	KLF2 IL10 TNF IL13 SCN2B EEF2 G6PD PDGFD HBG2 HBG1 CCNB1 CCND1 GPX2 MB CASP8 IL6 CSF3 BCL2 CD14 S100A9 S100A8 CYP1B1 ALOX5AP PTGS2 MT1X TNFAIP3 BAX PCNA SESN1 TP53INP1 CDKN1A MT1M MT1E MT1G MT1H MDM2 MT1B MT1A CRYAB GCH1 HMOX1 MT1F MT2A SLC7A11 SOD2 NR4A2 EGR1

GO:2001233	regulation of apoptotic signaling pathway	3.606	HERPUD1 IL7 TNF CSF2 BMP4 CXCL12 FAIM2 MAP2K5 SLC35F6 LCK HMGB2 APAF1 CASP8 FAS FASLG IL1B MMP9 BCL2 S100A9 S100A8 PTGS2 BCL2A1 TNFAIP3 IL1A ATF3 BAX TNFRSF10B GDNF MDM2 G0S2 HMOX1 HSPA1A INHBA SOD2 NR4A2 SCG2
